# Supplementary material for: Decarbonising the iron and steel sector for a 2 °C target using inherent waste streams
Source: Nat Commun. 2022 Jan 13;13:297. doi: 10.1038/s41467-021-27770-y (PMC8758725; doi:10.1038/s41467-021-27770-y)
Supplement: Supplementary file 1 — Supplementary Information [file 41467_2021_27770_MOESM1_ESM.pdf]

Supplementary Information

Decarbonizing the iron and steel sector for a 2 °C target using inherent waste streams

Yongqi Sun[1,2], Sicong Tian[2], Philippe Ciais[3], Zhenzhong Zeng[1]*, Jing Meng[4]*, Zuotai Zhang[1,5]*

[1]School of Environmental Science and Engineering, Southern University of Science and Technology, Shenzhen 518055, China

[2]School of Chemical Engineering, The University of Queensland, Brisbane, St Lucia, QLD 4072, Australia

[3]Laboratoire des Sciences du Climat et de l'Environnement, UMR 1572 CEA-CNRS UVSQ, 91191, Gif sur Yvette, France

[4]The Bartlett School of Construction and Project Management, University College London, London WC1E 7HB, UK

[5]The Key Laboratory of Municipal Solid Waste Recycling Technology and Management of Shenzhen City, Shenzhen 518055, China

* Correspondences: zengzz@princeton.edu; jing.j.meng@ucl.ac.uk; zhangzt@sustech.edu.cn

Supplementary notes

Supplementary note 1-Various treatment strategies for BFS based on the fundamental properties

For BFS, two final states will be obtained due to the individual cooling rates accompanying various practical cooling processes: a glassy state and crystalline state[1-6]. The critical cooling rate is an important parameter generally used to evaluate the crystallization ability or glass-forming ability of a high temperature liquid slag, i.e., the lowest cooling rate required to transform the liquid slag into a glassy state or the highest cooling rate required for crystal formation by structural relaxation of the slag[7,8]. If the liquid slag can be quickly cooled from a discharge temperature of 1550 °C to room temperature of 25 °C, a glassy state can be obtained. If the liquid slag is cooled slowly, crystals will form in the liquid slag, and therefore, a crystalline state will be obtained. Currently, there are three main ways to cool hot BFS.

The first way is to naturally cool high-temperature liquid BFS in a slag yard[9-11], and BFS treated in this way will finally be present in a crystalline state. As a result, both the thermal heat and material resources are wasted, and thus, this way is not considered in the present pathways.

The second way is to quench the slag by cool water[9-11], and thus, the slag can finally be collected in a glassy state, which can be further applied for cement production in replacement of $CaCO_3$ calcination[12-14]. In this way, the material resources of BFS are utilized while the thermal heat is wasted. This method is named BFS-Glassy/Water, representing that glassy BFS will be formed by water quenching. In addition to heat loss and water waste, this method has other disadvantages such as air pollution due to the release of H_2S and SO_2[9-11,15,16]. Herein, the case of BFS-Crystalline/Water (crystalline BFS obtained by water quenching) is not considered because this process will result in thermal heat loss and water consumption, while the obtained BFS is still in a crystalline state with resource recycling not totally achieved.

The third way is to quickly cool the slag using a dry agent instead of water to obtain a glassy state, during which the thermal heat is recovered. Due to the low thermal conductivities of liquid slag[9-11,17], a high-efficiency granulation step is required to break up the slag into small droplets and particles to increase the heat transfer area between the slag and the heat transfer agent. For this reason, numerous dry granulation methods are currently being developed globally, such as rotary cup atomizers (RCAs)[18-23], spinning disk atomizers (SDAs)[24-28] and rotary cylinder atomizers (RCLAs)[29-31], for the purpose of recovering the thermal heat and obtaining a glassy state of BFS for cement production.

Herein, two different results can be caused based on the technological levels of dry granulation, i.e., the hot slag is quickly cooled to obtain a glassy state, BFS-Glassy/Dry, or it is cooled slowly enough to obtain a crystalline state, namely BFS-Crystalline/Dry. The latter situation will degrade the further utilization of BFS for cement production. Using a dry granulation method, if a crystalline state is obtained, the thermal heat recovered can be greater since the crystal-forming process is exothermic where more energy will be released. To simplify the analysis, here it is assumed that the part of the energy release due to crystallization behaviours is 15% of the total sensible heat based on previous measurements[2,10,18]. Therefore, in this study, three treatment strategies are considered for the treatment of BFS: BFS-Glassy/Water, BFS-Glassy/Dry, and BFS-Crystalline/Dry (BFS-Crystalline/Water is excluded).

Supplementary note 2-Various treatment strategies for SS based on the fundamental properties

For SS, its crystallization ability is very strong due to the high basicity (mass ratio of CaO to SiO_2) and high "FeO" content, and therefore, it is very difficult to fully avoid the crystallization behaviour of SS because of its high liquidus temperature[32-36]. In other words, it is very challenging to obtain a glassy state of SS using a dry granulation method based on the current technological level, namely SS-Glassy/Dry; the utilization of SS- Glassy/Dry requires great advancements in granulation methods. Thus, there are only two strategies to be considered here. First, the high temperature liquid slag is cooled by dry granulation, and finally, a crystalline state is obtained, namely SS-Crystalline/Dry. Currently, the granulation methods developed for SS are quite limited[37]. However, through this method, the solid slag cannot be directly used as a raw material for cement production, and thus, the material resources of SS are wasted. On the other hand, for some special types of SS, their crystallization behaviour can be modified to improve the growth of target phases such as phosphorus-bearing phases[36,38-40]. The enriched phases are then separated from the base phases and the valuable minerals can be recycled. This method is named selective crystallization and phase separation (SCPS), through which part of the thermal heat can be recovered. The valuable resources, such as P, Fe and Ca, in the SS can be used after the SCPS process.

To further treat and utilize the crystalline SS, two situations are considered. First, the material resources after the dry granulation method, especially the CaO in the SS, are not considered, and as a result, only heat recovery is realized. Second, through advanced process design, it is possible to recycle the CaO in the SS for the flux in a steel plant or for the raw materials in a cement plant, as well as the utilization of Fe and P resources[41-46]. In this study, both situations are considered, and therefore, two options can be established based on dry granulation of SS,

namely SS-Crystalline/Dry and SS-Crystalline/Dry-R, where SS-Crystalline/Dry-R represents that the CaO in crystalline SS is further recycled.

Second, the liquid slag is assumed to be fully quenched to obtain a glassy state, namely, SS-Glassy/Water. However, like BFS treatment using water quenching, the thermal heat in the slag is wasted, and much water will be consumed, which will increase the cost of this method. More importantly, even using water quenching, SS can still exist in a partially crystalline state due to its high crystallization ability, namely SS-Crystalline/Water. In this case, both the thermal heat and material resources are wasted, and thus, SS-Crystalline/Water is not considered a promising option to be discussed in this study. Therefore, to analyse the practical pathways of SS utilization, three treatment strategies are constructed: SS-Crystalline/Dry, SS-Crystalline/Dry-R, and SS-Glassy/Water (SS-Crystalline/Water is excluded).

Supplementary note 3-Various technological schemes to engineer Pathways 5 and 6

In Pathways 5 and 6, the thermal heat in both BFS and SS is recovered. After energy recovery, BFS is in a glassy state, while SS is in a crystalline state, considering its strong crystallization ability. Regarding waste energy recovery, two types of methods can be applied, namely physical and chemical ones. Based on these methods, five technological schemes can be proposed, named Schemes 1-5.

For the physical method, because of the low thermal conductivities of high-temperature slag[9-11,17], the liquid slag should first be granulated into small droplets to increase the surface between the agent (air) and the slag, named Scheme 1. Therefore, the thermal heat in the slag can be quickly transferred to the agent (air). As a result, a high-temperature agent (air) can be obtained for further utilization, such as power generation, and at the same time, the slag is quickly cooled to obtain a glassy state to be used for cement manufacturing. Currently, a series of dry granulation methods are being developed including air blast and centrifugal methods such as RCAs, SDAs and RCLAs[18-31].

After granulation, the residual thermal heat transfer in the slag can be further transferred to the cool air, and then the thermal heat in the hot air can finally be transferred to steam for heat utilization. Thus, the physical method consists of three steps, namely air-slag granulation, air-slag heat transfer and air-steam heat transfer. Herein, to simplify the analysis, several assumptions are made. First, the granulation step takes place at 1550-1200 °C and 1600-1200 °C for BFS and SS, respectively, and an air flow with a temperature of 600 °C is obtained. The heat transfer efficiency between the air and liquid slag in this temperature range is assumed to be 70% to confirm the rapid cooling of the slag. In the second step, the full heat transfer between the slag and air occurs at 1200-100 °C and an air flow of 600 °C is obtained. The heat transfer efficiency between the air and solid slag in this step is assumed to be 80% since the glassy state has already been obtained, which can fully exchange heat with the air. Finally, the thermal heat

in the air is transferred to the steam, and a steam product with a final temperature of 200 °C is obtained.

For the chemical methods, the thermal heat in high-temperature slag acts as the heat source for endothermic reactions such as limestone decomposition, methane reforming and gasification and pyrolysis reactions[47-62]. The chemical methods, especially gasification and pyrolysis reactions, exhibit the advantages of high efficiency of heat transfer; production of valuable syngas such as CO, H_2 and CH_4; and integration of multiple sectors[9-11,17]. For energy recovery using chemical methods, the high-temperature slag still needs to first be granulated into small droplets or particles, and then for gasification or pyrolysis, the slag is mixed with fuel to provide thermal heat to support these reactions. Herein, the gasification method is analyzed due to these special advantages.

Regarding the gasification method, two key issues should be pointed out. The first is the temperature range for granulation. To confirm that a large part of the thermal heat is transferred to the gasification reaction to achieve a high energy efficiency, the granulation of BFS and SS is assumed to take place in a small temperature range, namely 1550-1350 °C and 1600-1400 °C, respectively. This will be realized based on the advancements of granulation technologies. The second issue is the granulation agent that is used. For the physical method, air is generally used, while for the chemical methods, in addition to air, CO_2 can be used considering further gasification reactions. For gasification, three agents can be employed, namely pure CO_2, pure H_2O (steam) and a mixing agent of CO_2 and H_2O. For CO_2 gasification or mixing agent gasification, a CO_2 agent is preferred for physical granulation because it is much easier for the heat in CO_2 to be used. Hot CO_2 can be directly mixed with room temperature CO_2 or H_2O to act as both a gasification agent and heat source for gasification. However, for air/slag granulation, a loss of energy efficiency will be caused since the thermal heat in air needs to be transferred to the gasification agent such as CO_2 or H_2O for further gasification reactions.

According to the granulation agent and gasification agent, heat utilization via chemical gasification can be divided into four schemes: air granulation + CO_2 gasification, air granulation + H_2O gasification, CO_2 granulation + CO_2 gasification and CO_2 granulation + CO_2/H_2O gasification. The scheme of air granulation+ CO_2/H_2O gasification is ignored since it does not present apparent advantages over the other schemes and a more complicated process is required. Here, it is assumed that for BFS and SS, similar energy recovery methods are used since if we use different energy recovery methods for the BFS and SS treatments, the situations will become so complex that the comparison and discussion will lose their meanings.

In summary, for the realization of Pathways 5 and 6, herein five schemes are constructed, namely the physical method, air granulation + CO_2 gasification, air granulation + H_2O gasification, CO_2 granulation + CO_2 gasification and CO_2 granulation + CO_2/H_2O gasification, which are denoted Scheme 1, Scheme 2, Scheme 3, Scheme 4 and Scheme 5, respectively, as summarized in Supplementary Fig. 20.

Supplementary note 4-Process descriptions and analysis methodology for Schemes 1-5

Scheme 1

The detailed process of Scheme 1 is sketched in Supplementary Fig. 21. Overall, it is divided into three steps: a first step of liquid slag granulation using cool air flow, a second step of full heat transfer between the solid slag and cool air flow, and a third step of heat transfer from the hot air to produce steam. As a result, steam and the solid slag are the main products of Scheme 1. Currently, most granulation methods have been developed for BFS[18-31], while those for SS are quite limited[37]. Therefore, in this section, the discussion focuses on BFS. For SS, it is assumed that similar technologies will be used considering future technological advancements.

In the first step, the high-temperature BFS is granulated into small droplets and quickly cooled to a glassy state by transferring the thermal heat to cool air with a high flow rate. The temperature of the BFS decreases from 1550 °C to 1200 °C, and the air temperature increases from 25 °C to 600 °C. Due to the requirement of rapid cooling of the liquid slag, we assume that the heat transfer efficiency between the slag and air is 70%. The mass and energy balances of step 1 are expressed by Eq. (1):

$$m_{BFS} * C_{p,BFS}(T_{BFS,2} - T_{BFS,1}) = m_{a,1} * C_{p,a} * (T_{a,2} - T_{a,1}) \quad (1)$$

where m_{BFS} and $m_{a,1}$ represent the masses of the BFS and air, respectively; $C_{p,BFS}$ and $C_{p,a}$ represent the heat capacities of the BFS and air, respectively; and $T_{BFS,1}$, $T_{BFS,2}$ and $T_{a,1}$, $T_{a,2}$ represent the temperature points of the BFS and steam before and after heat transfer, respectively.

In the second step, the heat transfer between the hot glassy slag and cool air will fully take place, where a fluidized bed can be used with a heat transfer efficiency of 80%. The temperature of the glassy slag will further decrease from 1200 °C to 100 °C, while the air temperature will increase from 25 °C to 600 °C. The mass and energy balances of step 2 are expressed by Eq. (2):

$$m_{BFS} * C_{p,BFS}(T_{BFS,3} - T_{BFS,2}) = m_{a,2} * C_{p,a} * (T_{a,2} - T_{a,1}) \quad (2)$$

where m_{BFS} and $m_{a,2}$ represent the masses of the BFS and air, respectively; $C_{p,BFS}$ and $C_{p,a}$ represent the heat capacities of the BFS and air, respectively; and $T_{BFS,2}$, $T_{BFS,3}$ and $T_{a,1}$, $T_{a,2}$ represent the temperature points of the BFS and steam before and after heat transfer, respectively.

In the third step, full heat transfer from the obtained hot air at 600 °C to room temperature water (25 °C) will occur to produce steam, with a heat transfer efficiency of 80%. In this step, the temperature of the air will decrease from 600 °C to 100 °C, while that of the steam will increase from 25 °C to 200 °C. The mass and energy balances of step 3 are expressed by Eq. (3):

$$(m_{a,1} + m_{a,2}) * C_{p,a} * (T_{a,2} - T_{a,3}) = m_s * [C_{p1,s}(T_{s,2} - T_{s,1}) + \Delta H_s + C_{p2,s}(T_{s,3} - T_{s,2})] \quad (3)$$

where $m_{a,1} + m_{a,2}$ and m_s represent the masses of hot air and steam, respectively; $C_{p,a}$, $C_{p1,s}$ and $C_{p2,s}$ represent the heat capacities of hot air, water at 25-100 °C and steam at 100-200 °C, respectively; ΔH_s represents the latent heat of steam at 100 °C; and $T_{a,2}$, $T_{a,3}$ and $T_{s,1}$, $T_{s,2}$, $T_{s,3}$ represent the temperature points of air and steam, respectively.

Regarding the SS treatment, two other important issues different from those in the BFS treatment should be highlighted. First, the temperature range of the first step for SS is different from that from BFS, i.e., it takes place at 1600-1200 °C. Second, due to the stronger crystallization ability, SS is finally obtained in a crystalline state instead of a glassy state, and 15% more of the thermal energy will be recovered from crystal precipitation.

Scheme 2

Scheme 2 represents a gasification method using air as the granulation agent and CO_2 as the gasification agent, as sketched in Supplementary Fig. 22. The process of Scheme 2 will be expressed in detail here, because this is the first scheme using a gasification reaction to treat the

high-temperature slag; Schemes 3-5 follow similar processes to Scheme 2, where only the granulation or gasification agent is adjusted. The treatment process of BFS is also detailed here, while for SS, a similar process is employed.

In the first step of Scheme 2, the high-temperature liquid BFS is granulated into small droplets and then glassy particles in a granulator, and additionally, the thermal heat in the BFS is transferred to the cool air. To ensure that a large part of the thermal heat is used for the gasification reaction, the slag temperature in this step decreases from 1550 °C to 1350 °C while the air temperature increases from 25 °C to 600 °C. Because of the severe requirement of slag cooling, it is assumed that the heat transfer efficiency in this step is 70%. In the second step, the gasification reaction takes place in the temperature range of 1350-800 °C. In this step, the gasification fuel will react with CO_2 to produce syngas composed of CO, H_2, CH_4, CO_2 and H_2O using the thermal heat in the slag. After gasification, the temperature of the BFS will decrease from 1200 °C to 800 °C and the hot syngas at 800 °C is produced.

The third step is related to the deep utilization of the residual heat, including the 600 °C hot air from the first step and the 800 °C BFS and syngas from the second step. Three types of heat utilization methods can be selected. First, the heat can be used to heat the gasification fuel to a gasification temperature of 800 °C. Second, the heat can be used to heat the CO_2 gasification agent to 800 °C. Third, the heat can also be used for steam production at a temperature of 200 °C. In the present schemes, the excess energy of the BFS and hot syngas is used for both heating the gasification agent and steam production. It is assumed that the final temperature of the BFS and syngas is 100 °C. It should be pointed out that the heat transfer efficiency in all these methods of heat utilization is 80%, considering the full heat transfer between different agents.

The energy balance of the whole process is expressed by Eq. (4) as follows:

$$Q_{fuel,s} + Q_{fuel,l} + Q_{agent,s} + Q_{agent,l} + Q_{BFS} = Q_{loss} + Q_{syngas,s} + Q_{syngas,l} + Q_{steam}$$

(4)

where $Q_{fuel,s}$ and $Q_{fuel,l}$ represent the sensible and latent heat of the fuel, respectively; $Q_{agent,s}$ and $Q_{agent,l}$ represent the sensible and latent heat of the gasification agent, respectively; Q_{BFS} represents the thermal heat in the BFS; Q_{loss} represents the heat loss in various steps; $Q_{syngas,s}$ and $Q_{syngas,l}$ represent the sensible and latent heat of the syngas, respectively; and Q_{steam} represents the thermal energy in the steam product.

For the gasification agent,

$$Q_{agent,s} + Q_{agent,l} = 0 \tag{5}$$

For the gasification fuel,

$$Q_{fuel,s} + Q_{fuel,l} = HHV_{fuel} \tag{6}$$

where HHV_{fuel} (MJ/kg) is the higher heating value of the gasification fuel, which can be estimated based on the chemical compositions, and is expressed as follows[63,64]:

$$HHV_{fuel} = 0.3491[C] + 1.1783[H] - 0.1034[O] \tag{7}$$

where $[C]$, $[H]$ and $[O]$ represent the weight ratios of C, H and O elements in the fuel.

For the syngas,

$$Q_{syngas,s} + Q_{syngas,l} = HHV_{syngas} \tag{8}$$

where HHV_{syngas} (MJ/Nm^3) is the higher heating value of syngas, which can be estimated based on the syngas components, and is expressed as follows[63,64]:

$$HHV_{syngas} = 12.75[H_2] + 12.63[CO] + 39.82[CH_4] \tag{9}$$

where $[H_2]$, $[CO]$ and $[CH_4]$ represent the volume ratios of H_2, CO and CH_4 in the dry syngas.

Combining Eqs. (4)-(9), Eq. (10) can be derived as follows:

$$HHV_{syngas} - HHV_{fuel} + Q_{steam} = Q_{BFS} - Q_{loss} \tag{10}$$

In Eq. (10), the energy loss can be divided into four parts, namely, the energy loss in the granulation step at 1550-1350 °C, the energy loss during the cooling process of BFS from 800 °C to 100 °C, the energy loss of syngas cooling from 800 °C to 100 °C and the latent heat loss of the steam in the syngas. The energy loss can be expressed by Eq. (11):

$$Q_{loss} = Q_{l1,1550-1350^oC\,BFS} + Q_{l2,800-100^oC\,BFS} + Q_{l3,800-100^oC\,syngas} + Q_{l4,steam\ in\ syngas}$$

(11)

Combining Eqs. (10) and (11), an equation expressing the total energy balance in this system can be derived as follows:

$$HHV_{syngas} - HHV_{fuel} + Q_{steam} = Q_{BFS} - (Q_{l1,1550-1350^oC\,BFS} + Q_{l2,800-100^oC\,BFS} + Q_{l3,800-100^oC\,syngas} + Q_{l4,steam\ in\ syngas})$$

(12)

Another important equation determining the energy balance is related to the production of steam where the energy comes from three parts, namely, the 600 °C air, 800 °C BFS and 800 °C syngas. This equation is expressed as follows:

$$\eta_1 * \eta_2 * Q_{1550-1350^oC\,BFS} + \eta_3 * Q_{800-100^oC\,BFS} + \eta_4 * (Q_{800-100^oC\,syngas} + Q_{L',steam\ in\ syngas}) = Q_{steam} \quad (13)$$

where $Q_{1550-1350^oC\,BFS}$ and $Q_{800-100^oC\,BFS}$ represent the sensible heat of the BFS in the temperature ranges of 1550-1350 °C and 800-100 °C, respectively; $Q_{800-100^oC\,syngas}$ represents the sensible heat of the syngas in the temperature range of 800-100 °C; $Q_{L',steam\ in\ syngas}$ represents the latent heat of the steam in the syngas; and η_1, η_2, η_3 and $\eta_4$represent the energy efficiencies of air-BFS heat transfer at 1550-1350 °C, air-steam transfer at 600-100 °C, air-BFS heat transfer at 800-100 °C and syngas-steam heat transfer at 800-100 °C, respectively.

Eqs. (12) and (13) are the main equations determining the mass and energy balances of these schemes, through which the mass ratio of fuel/BFS, and the yields of valuable products including slag, steam and syngas can be obtained. In this study, the sensible heat of the syngas is calculated based on a linear summation of the gases composing in the syngas, expressed by Eq. (14):

$$Q_{800-100^oC\,syngas} = \chi_{CO}Q_{CO} + \chi_{H_2}Q_{H_2} + \chi_{CH_4}Q_{CH_4} + \chi_{CO_2}Q_{CO_2} + \chi_{H_2O}Q_{H_2O}$$

(14)

2here χ_{CO}, χ_{H_2}, χ_{CH_4}, χ_{CO_2} and χ_{H_2O} represent the weight ratios of CO, H_2, CH_4, CO_2 and H_2O, respectively, Q_{CO}, Q_{H_2}, Q_{CH_4}, Q_{CO_2} and Q_{H_2O} represent the sensible heat of CO, H_2, CH_4, CO_2 and H_2O, respectively.

The sensible heat of various gases in the temperature range of 800-100 °C is calculated based on their individual heat capacities by Eq. (15):

$$Q_{800-100^oC} = \int_{100^oC}^{800^oC} C_{p,T}dT \quad (15)$$

Regarding SS, a similar process is performed, where the temperature of the first step changes to 1600-1400 °C, the temperature of the second step changes to 1400-800 °C, and SS is obtained in a crystalline state instead of a glassy state.

Another important issue should be highlighted here, i.e., how to utilize the latent heat of the steam in the syngas. With regard to this part of energy, two methods can be used. The first method is that the latent heat of the steam is transferred to the cool fuel or gasification agent with a transfer efficiency of 80%. Another method is that this part of heat is totally lost since the discharge temperature of the syngas is assumed to be 100 °C, which is the condensation temperature of steam under 1 atm. In this study, the first method is discussed to maximize the energy efficiency of the different schemes.

Scheme 3

Scheme 3 represents a gasification method using air as the granulation agent and H_2O as the gasification agent, as sketched in Supplementary Fig. 23. The differences between Scheme 3 and Scheme 2 are that in the second step, a steam flow with a temperature of 200 °C is used as the gasification agent, while in the third step, the sensible heat of the 800 °C syngas and BFS is mainly used to heat room temperature water to produce 200 °C steam and heat the gasification

fuel; the steam produced will be further used as the gasification agent. It is assumed that the heat transfer efficiencies in each step are the same as those in Scheme 2.

For the energy and mass balances of Scheme 3, Eq. (12) and Eq. (13) are also used to determine the mass ratio of fuel/BFS and the yields of the products, including steam and syngas. In these equations, Q_{BFS} , $Q_{l1,1550-1350^{o}C\,BFS}$ and $Q_{l2,800-100^{o}C\,BFS}$ are the same, which are only determined by BFS, and HHV_{fuel} , $Q_{l4,steam\ in\ syngas}$ and Q_{steam} are related only to the amounts of fuel and steam; however, HHV_{syngas}, $Q_{l3,800-100^{o}C\,syngas}$ and $Q_{l4,steam\ in\ syngas}$ are totally different not only because of the components of the syngas but also because of the syngas yields in different schemes. Another difference in Eq. (4) between Scheme 3 and Scheme 2 is that for Scheme 3, Q_{steam} includes the thermal heat both in the steam product and in the steam agent for the gasification reaction.

Scheme 4

Scheme 4 represents a gasification method using CO_2 as the granulation agent and CO_2 as the gasification agent, as sketched in Supplementary Fig. 24. Regarding the detailed process, the differences between Scheme 4 and Scheme 2 are that slag granulation is realized using a high flow rate of CO_2 gas and that part of the hot CO_2 produced from the first step will be directly used as a gasification agent in the second step. Thus, the energy loss can be decreased because the heat transfer step between the granulation agent and the gasification agent will be avoided. Therefore, in Eq. (12) and Eq. (13), the energy loss for the first step, $Q_{l1,1550-1350^{o}C\,BFS}$, and the energy for steam production, $\eta_1 * \eta_2 * Q_{1550-1350^{o}C\,BFS}$, will vary. As a result, these two types of energy should be modified based on the amount of gasification fuel, with a mole ratio of fuel/CO_2 of 2:1; however, for other types of energy in the whole process, the same calculation equations are used.

Scheme 5

Scheme 5 represents a gasification method using CO_2 as the granulation agent and a mixing agent of CO_2/H_2O as the gasification agent, as sketched in Supplementary Fig. 25. Regarding the detailed process, the differences are that, the slag granulation is realized using a high flow rate of CO_2 gas and that part of the hot CO_2 is directly mixed with steam to work as the gasification agent; therefore, less heat loss is realized. Similar to Scheme 4, the energy loss in the first step, $Q_{l1,1550-1350^oC\,BFS}$, and the energy for steam production, $\eta_1 * \eta_2 * Q_{1550-1350^oC\,BFS}$, will vary. These two values will be adjusted based on the amount of fuel a fuel/CO_2/H_2O mole ratio of 1:1:1.

Supplementary note 5-Cost-benefit analysis per tonne of BFS and SS

Based on the process analysis, the costs and benefits of the different schemes can be obtained. There are three types of costs, namely capital costs, operating costs and CO_2 price caused by energy consumption. Overall, the benefits are composed of two types, namely product benefits and revenues by CO_2 avoided due to energy recovery and emission reduction. The operating costs are further divided into maintenance costs, labour costs, resource costs and energy costs, while the products obtained in these schemes include cooled BFS and SS, steam and syngas. Regarding CO_2 price, both costs and benefits will be considered due to energy consumption and energy recovery.

Several significant issues should be pointed out here regarding the cost-benefit estimation. First, both BFS and SS are considered, and thus, in the final results, we can obtain the average economic benefits per tonne of crude steel, where the Pig iron/Crude steel BFS/Pig iron and SS/Crude steel ratios are selected to be 0.71, 0.26 and 0.13, respectively.

Second, four scales of cost and benefit analyses can be calculated, namely the level of per tonne of BFS and SS, the level of per tonne of crude steel, the level of a steel plant and the level of the global iron and steel sector (Supplementary Fig. 19). It should be highlighted here that cost and benefit values in the literature are adjusted considering global income and labour levels since most literature focuses only on the level of one single country or area[58,65-67]. For the plant level analyzed in detail in this study, we assume that in a steel plant, 1 Mt crude steel is produced annually.

Third, here US dollars (US$) are used to calculate the costs and benefits, and if other currencies are used in the literature, they are converted based on the exchange rates.

Fourth, the revenue in the cement industry due to the utilization of BFS and SS in cement manufacturing is not included here since the economic analysis focuses on the iron and steel sector. However, for the potential analysis of various pathways, this part is included since we

discuss the emission reduction ratio to the 2 °C target requirements on a larger sectoral scale composed of various sectors.

As mentioned above, in the present study, five schemes are considered for the energy recovery and resource recycling of BFS and SS. Here, the cost-benefit analysis of BFS is first discussed. In Scheme 1, physical granulation is used for energy recovery, which includes three steps, namely slag granulation, heat transfer between air and slag and heat transfer between hot air and steam. The estimation of Scheme 1 is of special significance since for the other schemes, generally a similar granulation will necessarily account for the first step. Norgate et al.[6] performed economic evaluations of the energy recovery of BFS using a physical granulation method based on industrial experiments. The parameters related to the capital costs are summarized in Supplementary Table 13 (where an exchange rate of 0.7 US$=1 Australian $ was used). It should be pointed out here that the CO_2 price is 0 since no fossil fuels are used directly and the energy required is mainly electrical energy. Considering the global average price level, the parameters are adjusted here. Thus, on average, for a steel plant, US$10.52 is the cost per tonne of BFS.

Regarding the benefits of Scheme 1, herein, three kinds of benefits will be generated, namely glassy slag, steam and CO_2 avoided benefits. Thus, several points related to these benefits should be highlighted here. First, for the glassy slag, recent information from the USGS is used[67]. This information is from a wide range of different countries, and herein, an average price of US$75 per tonne of BFS is used.

Second, for the benefits of steam, the price is estimated based on the latent and sensible heat carried by the steam. Herein, it is assumed that the steam is produced by natural gas combustion with overall combustion and heat transfer efficiencies of 80%. The price of natural gas is estimated based on the energy carried, with a value of US$6 per MMBtu[68].

Third, the benefits of CO_2 avoided cover a wide range of different countries and areas worldwide, and here, considering the global level, an average value of US$30 per tonne of CO_2 emissions is applied[69-73]. Other important parameters related to the cost-benefit analysis are listed in Supplementary Tables 5 and 13.

Based on the aforementioned discussions, the cost-benefit analysis of Scheme 1 could be obtained, as detailed in Supplementary Table 6. The average cost of Scheme 1 is US$10.52 per tonne of BFS, while the average benefit is US$85.16 per tonne of BFS. As a result, the net revenue of Scheme 1 is US$74.64 per tonne of BFS, representing a great potential benefit not fully developed globally.

In Schemes 2-5, a chemical gasification method is used to recover the waste heat of BFS, which is mainly composed of a quick granulation process and a gasification reaction. It is assumed that the capital cost does not change greatly since for the current level, a reactor composed of a liquid slag granulator and chemical gasification reactor is expected to be developed[47,74]. Regarding the operating costs, it is assumed that the labour and maintenance costs do not change compared with those in Scheme 1, while the energy costs are assumed to increase by 20% since a new step of chemical gasification is used. However, compared to Scheme 1, a new kind of cost is necessary, namely material costs due to the utilization of coal, biomass and sludge. If coal is utilized, two kinds of costs will be incurred, i.e., fuel costs based on the market price and CO_2 costs due to the utilization of coal, a traditional fossil fuel. However, if biomass or sludge is applied, resource costs can be avoided since they are common solid wastes, and additionally, CO_2 costs can also be avoided since they are carbon-neutral materials. In fact, the avoidance of resource costs and CO_2 emission costs accounts for the important advantages of Schemes 2-5.

Regarding the benefits of Schemes 2-5, in addition to glassy slag, steam and CO_2 avoided, a new type of benefit will be produced, namely valuable syngas composed of CO, H_2, CH_4 and CO_2. Regarding the estimation of syngas prices, two methods can be used. On the one hand, the

sensible heat and latent heat of syngas are directly transformed into the corresponding energy of natural gas and thus the benefits. On the other hand, syngas is one kind of product from the energy recovery of BFS, similar to the steam product. In the present study, we estimate the steam price by assuming that it is produced by the combustion of natural gas with an energy efficiency of 80%. Thus, here, it can be assumed that syngas can be produced using natural gas with the same efficiency of 80% since in this study we compare the benefits of these various schemes. Therefore, in this study we use the second method to estimate the value of syngas. Another important issue is the gasification fuel used, coal, biomass or sludge, since if biomass or sludge is used, both the material costs and CO_2 penalty can be avoided.

The results of the cost-benefit analysis for the BFS energy recovery using Schemes 2-5 are shown in Supplementary Tables 7-10. As can be observed, regarding the costs, if coal is used, the costs will increase from US$10.52 per tonne of BFS for Scheme 1 to ~ US$13.0 per tonne of BFS in Schemes 2-5. However, if solid wastes are used such as biomass or sludge, the costs only slightly increase to ~ US$11 per tonne of BFS because the material costs of coal and thus a part of the CO_2 costs are avoided. The average benefit increases from US$85.16 per tonne of BFS in Scheme 1 to ~ US$88.6 per tonne of BFS in Schemes 2-5. It can also be noted that there is not much difference regarding whether coal or biomass and sludge are used since there is only a small difference between the CO_2 avoided benefits for the different schemes. Based on the costs and benefits, the net revenue per tonne of BFS increases slightly from US$74.64 in Scheme 1 to ~ US$75.5 in Schemes 2-5 when coal is used. However, if biomass or sludge is used, the net revenue per tonne of BFS increases to ~ US$77.9, mainly due to the reduction in fuel costs.

Regarding the energy recovery and resource recycling of SS, several different issues should be pointed out compared with those of BFS. First, there is currently no successfully commercialized granulation process for SS. Therefore, it is assumed that the granulation cost

of SS is similar to that of BFS based on future technological advancement, i.e., the capital costs and the operating costs, including labour, maintenance and energy costs, do not change, while only the resource costs slightly change when the amount of fuel changes for SS energy recovery, as summarized in Supplementary Tables 6-10. Second, regarding the final products, SS is in a crystalline state in these schemes because of its stronger crystallization ability caused by the high basicity and "FeO" content. Although crystalline SS has a smaller utilization value, it also covers a wide range of US$3-10 per tonne of SS. Here, an average level of US$5.88 per tonne of crystalline SS is applied. If very advanced granulation of SS is developed in the long term, a glassy SS can be produced, and a much larger revenue is expected to be created due to its high value.

The results of the cost-benefit analysis for SS treatment via Schemes 1-5 are also detailed in Supplementary Tables 6-10, respectively. As can be observed, regarding the costs, if coal is used, the costs will increase from US$10.52 per tonne of SS in Scheme 1 to ~ US$14.0 per tonne of SS in Schemes 2-5. However, if solid wastes are used, such as biomass or sludge, the cost only slightly increases to ~ US$10.8 per tonne of SS because a large amount of the fuel costs and thus CO_2 costs are avoided. The average benefit increases from US$19.0 per tonne of SS in Scheme 1 to ~ US$21.8 in Schemes 2-5. It can also be noted that there is not much difference regarding whether coal or waste is used since the CO_2 avoided revenues for the different schemes are very limited. Based on the costs and benefits, the net revenue per tonne of SS decreases slightly from US$8.48 in Scheme 1 to ~ US$8.0 in Schemes 2-5 when coal is used. However, if solid waste, such as biomass or sludge, is used, the net revenue increases to ~ US$11.1 per tonne of SS mainly because a large amount of the fuel costs and the CO_2 emission penalty will be avoided.

Supplementary note 6-Methodology to obtain the heat capacities of BFS and SS

There are two methods to obtain the heat capacities of slag. The first way is to calculate them based on the individual heat capacities of the oxides in the slag following several steps. First, these values can be directly collected based on FactSage software, which is commonly used for slag thermodynamic calculations[75]. Second, the heat capacities of these oxides are summed linearly based on their weight ratios, and thus, the heat capacities of BFS and SS can be derived. The chemical compositions of BFS and SS cover wide ranges due to the various operational parameters and iron ores in different steel plants. Herein, a series of studies and reviews are referred to, and a typical composition is summarized[47-62,76-78], as detailed in Supplementary Table 4. To simplify the calculation process, here, only the main components of CaO, SiO_2, MgO, Al_2O_3, Fe_2O_3 and FeO are considered, while the minor elements are ignored.

The second way is to directly calculate the heat capacities using the FactSage equilibrium mode. The results of these two methods are detailed in Supplementary Table 4. First, the heat capacities of SS are generally lower than those of BFS due to the relatively smaller heat capacities of Fe_2O_3 and FeO compared to those of CaO, SiO_2, MgO, and Al_2O_3. Moreover, it can be seen that there are few differences in the results calculated by these two methods, which can both be used to estimate the heat capacities BFS and SS. It should be pointed out that these calculated values are also in good agreement with the experimental results for some particular slag reported in previous studies[79-81], which also proves the reasonability of the present methodology.

Supplementary note 7-Control of gasification conditions for gasification equilibrium calculation

For the waste energy recovery from BFS and SS using a gasification method, there are three types of agents that can be used, namely, CO_2, H_2O (steam) and a mixture of CO_2 and H_2O. For the gasification methods, the important factors determining the results are the gasification fuel used, gasification temperature and fuel/agent ratio.

Regarding the gasification fuel, two types are considered here, namely, coal and solid wastes, including biomass and sludge. Generally, the content of fixed carbon in coal is much higher than that in biomass and sludge[47-62,82-84], and thus, more syngas can be produced. However, the utilization of biomass and sludge has the advantages of emission reduction and waste treatment since they are carbon-neutral solid wastes. The chemical compositions of coal, biomass and sludge are very different[47-62,82-84], such as the C, H and O concentrations and the ash/moisture contents. Here, to simplify the calculations and analysis, a typical chemical composition is selected, as detailed in Supplementary Table 4. In this study, only C, H and O elements are considered, while minor elements such as S and N are ignored.

Regarding the reaction temperature, in fact, the energy recovery from the slag occurs in a wide temperature range, and therefore, gasification will take place in this temperature range. To simplify the calculations and analysis, the lowest temperature of the slag is taken as the operational gasification temperature. Herein, 800 °C is used as the gasification temperature to ensure that a large part of the thermal heat is recovered for the gasification reaction.

Two additional important issues need to be highlighted here. The first is that the presence of some particular slag can change the yields of the final products, as proven in previous studies[47-62]. In detail, low-valence iron, Fe^{2+}, is present in SS, as shown in Supplementary Table 4, which can react with CO_2 and H_2O to produce CO and H_2, respectively. Compared to that of SS, the effect of BFS on the gasification products is quite limited. To simplify the calculations, here,

the effect of BFS and SS on the syngas yields is ignored. The yield of syngas is calculated using FactSage software[75]. The second issue is that, in addition to equilibrium syngas yields, the presence of BFS and SS can also change the gasification kinetics, i.e., the components in the slag, such as Ca and Fe elements, can act as positive catalysts for gasification, as proven in previous studies[47-62]. This accounts for another advantage of the integration of slag energy recovery with gasification reactions. In this study, the effect of BFS and SS on gasification kinetics is not considered since it does not affect the mass and energy balances in this system.

To quantify the gasification results, several parameters are defined, including the total syngas yield, CO content, H_2 content, carbon efficiency and hydrogen efficiency. There can actually be two types of the former three parameters depending on whether the steam in the syngas is considered. Herein, both values are calculated, and we mainly discuss the characteristics of dry syngas without steam since at room temperature, steam in syngas will condense into liquid water. Thus, four kinds of gases are mainly considered in the syngas, namely, CO, H_2, CH_4 and CO_2. The total yield of dry syngas (*Vs*), CO content (η_{CO}) and H_2 content (η_{H_2}) are defined by Eq. (16), Eq. (17) and Eq. (18), respectively. In addition to these three parameters, we also used the carbon efficiency (*CE*) and hydrogen efficiency (*HE*) to estimate the gasification efficiencies by means of the transformation efficiency of C and H from the gasification fuel to the syngas, which are defined by Eq. (19) and Eq. (20), respectively:

$$Vs = V_{CO} + V_{H_2} + V_{CH_4} + V_{CO_2} \quad (16)$$

$$\eta_{CO} = \frac{V_{CO}}{Vs} * 100\% \quad (17)$$

$$\eta_{H_2} = \frac{V_{H_2}}{Vs} * 100\% \quad (18)$$

$$CE = \frac{n_{C,CO} + n_{C,CH_4}}{n_{C,fuel}} * 100\% \quad (19)$$

$$HE = \frac{n_{H,H_2} + n_{H,CH_4}}{n_{H,fuel}} * 100\% \quad (20)$$

where Vs, V_{CO}, V_{H_2}, V_{CH_4} and V_{CO_2} represent the volumes of total syngas, CO, H_2, CH_4 and CO_2, respectively; η_{CO} and η_{H_2} represent the contents of CO and H_2 in the syngas, respectively; CE and HE represent the carbon efficiency and hydrogen efficiency during gasification, respectively; $n_{C,CO}$, n_{C,CH_4} and $n_{C,fuel}$ represent the moles of carbon in CO, H_2 and gasification fuel, respectively; and $n_{H,CO}$, n_{H,CH_4} and $n_{H,fuel}$ represent the moles of hydrogen in CO, H_2 and gasification fuel, respectively.

Supplementary note 8-Selection of gasification conditions based on the results of gasification calculations

The CO_2 gasification results with varying fuel/agent ratios from 0.5 to 10 are detailed in Supplementary Table 14. As can be observed, at 800 °C with an increasing amount of CO_2, the yield of CO continuously increases, while those of H_2 and CH_4 continuously decrease. As a result, the carbon efficiency continuously increases, while the hydrogen efficiency decreases. It can be noted that the carbon efficiency can reach values higher than 1.0 because the carbon in CO_2 is transformed into CO when the CO_2/fuel ratio is higher than 1.0. Due to the increasing residual CO_2 in the syngas with an increasing amount of CO_2, the H_2 content in the syngas continuously decreases. However, with an increasing amount of CO_2, the CO content first remarkably increases due to the enhanced reaction between CO_2 and fixed carbon and then continuously decreases.

A discussion of the yields of these gases can be conducted on the basis of several fundamental reactions[60,85,86]. These fundamental reactions include organic pyrolysis (Eq. 21), char transformation (Eq. 22), the Boudouard reaction (BD, Eq. 23), primary water gas (PWG, Eq. 24), water-gas shift (WGS, Eq. 25), methane formation (MF, Eq. 26), methane steam reforming (MSR, Eq. 27) and methane CO_2 reforming (MCR, Eq. 28). We can also observe that, using CO_2 as the gasification agent, the yield of CO is greatly larger than those of CO and CH_4 due to the dominant reactions of Eq. (23) and Eq. (28). Most importantly, we can see that as the amount of CO_2 increases to more than 1.5 moles, the residual carbon disappears due to the enhanced reaction of Eq. (23). Here, to confirm the full gasification of fuel and to ensure a low cost of syngas separation after gasification, a CO_2/fuel ratio of 2:1 is employed.

$$\mathrm{CH_{1.6}}O_{0.7}N_{0.008} \rightarrow aCO + bH_2 + cCH_4 + dCO_2 + eH_2O + fNH_3 + gNO + hNO_2 + Char + \ldots\ldots \quad (21)$$

Char transformation: $Char \rightarrow aC + bCO + cH_2 + dCH_4 + eCO_2 + fH_2O\ldots\ldots$ (22)

BD: $C(s) + CO_2\,(g) = 2CO\,(g),\ \Delta H = 168.9\ \mathrm{kJ/mol}$ (23)

PWG: $C(s)+H_2O(g)=CO(g)+H_2(g), \Delta H$=135.8 kJ/mol (24)

WGS: $CO(g)+H_2O(g)=CO_2(g)+H_2(g), \Delta H$= -33.1 kJ/mol (25)

MF: $C(s)+2H_2(g)=CH_4(g), \Delta H$= -91.3kJ/mol (26)

MSR: $CH_4(g)+H_2O(g)=CO(g)+3H_2(g), \Delta H$= 227.1kJ/mol (27)

MCR: $CH_4(g)+CO_2(g)=2CO(g)+2H_2(g), \Delta H$=260.2kJ/mol (28)

Regarding H_2O gasification, the results with varying H_2O/fuel ratios from 0.5 to 10 are detailed in Supplementary Table 15. As can be observed, with an increasing amount of H_2O, the yield of H_2 continuously increases due to the enhancement of Eqs. (24), (25), (27) and (28). The fixed carbon disappears at 1.0 mole H_2O. Thus, with an increase in the amount of H_2O from 0.5 to 1.0 mole, the CO gas increases, and with a further increasing amount of H_2O, the CO yield decreases due to the enhancement of Eq. (25). With an increasing amount of H_2O, the CH_4 yield decreases, which agrees with that under CO_2 gasification. The yields of CO and H_2 are comparable when H_2O is used as the gasification agent. It can be observed that the carbon efficiency continuously decreases while the hydrogen efficiency increases. The hydrogen efficiency is much larger than 1.0 because hydrogen is transformed from H_2O into H_2 and CH_4 gases. Similarly, to simplify the calculations and to compare the results with those under CO_2 gasification, a H_2O/fuel ratio of 2:1 is employed.

In addition to CO_2 gasification and H_2O gasification, another choice is to use a mixing agent of CO_2 and H_2O, which can be used to modify the components of syngas and the reaction kinetics in a target direction. To ensure gasification at a similar level and to simplify the discussion of different strategies, the total molar amount of CO_2 and H_2O is assumed to be 2.0, and the mole ratio of CO_2 to H_2O varies from 0.1:1.9 to 1.9:0.1. The results are detailed in Supplementary Table 16. As can be observed, when the mixing agent is used, there is no fixed carbon remaining. With an increasing CO_2/H_2O ratio, the yield of CO gas continuously increases, while that of H_2 gas continuously decreases. The yields of CO and H_2 are comparable, while the yield of CH_4 is

much lower, and slightly decreases with an increasing CO_2/H_2O ratio. As a result, the carbon efficiency gets continuously increased and the hydrogen efficiency continuously decreases with an increasing CO_2/H_2O ratio. When the ratio is larger than 0.6/1.4, both efficiencies are larger than 1.0 due to the carbon transformation from CO_2 and the hydrogen transformation from H_2O. Therefore, in this study, a CO_2/H_2O mole ratio of 1:1 is employed, and as a result, the final mole ratio of fuel/CO_2/H_2O is selected to be 1:1:1.

In summary, for the gasification method using different agents including CO_2, H_2O and a mixture of CO_2/H_2O, the agent/fuel mole ratios are all 2:1, and for the CO_2/H_2O mixture, a CO_2/H_2O mole ratio of 1:1 is employed.

Supplementary Tables

Supplementary Table 1-Product yields for various schemes

Products-BFS		Scheme 1	Scheme 2	Scheme 3	Scheme 4	Scheme 5
Steam Yield	kg/tonne of slag	399.87	324.43	298.17	321.01	325.49
Syngas Yield	Nm^3/tonne of slag		68.17	57.95	69.54	59.95
CO	Nm^3/tonne of slag		41.02	14.92	41.84	15.44
H_2	Nm^3/tonne of slag		2.19	33.33	2.24	34.48
CH_4	Nm^3/tonne of slag		0.00	0.03	0.00	0.03
CO_2	Nm^3/tonne of slag		24.96	9.66	25.46	10.00
Products-SS		Scheme 1	Scheme 2	Scheme 3	Scheme 4	Scheme 5
Steam Yield	kg/tonne of slag	455.58	322.74	285.06	317.83	324.25
Syngas Yield	Nm^3/tonne of slag		110.85	94.23	113.08	97.49
CO	Nm^3/tonne of slag		66.70	24.27	68.05	43.80
H_2	Nm^3/tonne of slag		3.57	54.20	3.64	26.17
CH_4	Nm^3/tonne of slag		0.00	0.05	0.00	0.03
CO_2	Nm^3/tonne of slag		40.58	15.72	41.40	27.50

Supplementary Table 2-Global economic revenues of the various schemes (US$, Billion)

Yr	Scheme 1	Scheme 2	Scheme 3	Scheme 4	Scheme 5
2020	27.01	28.72	28.68	28.74	28.72
2035	32.42	34.48	34.43	34.50	34.48
2050	38.00	40.41	40.36	40.44	40.41

Supplementary Table 3-Predictions of products in the iron and steel sector from 2020 to 2050

Year	2008	2009	2010	2011	2012	2013	2014	2015	2016	2017
Crude steel (Mt)	1343	1239	1433	1538	1560	1650	1669	1620	1627	1690
Pig iron (Mt)	949	933	1034	1104	1123	1207	1185	1158	1167	1180
Pig iron/Crude steel	0.71	0.75	0.72	0.72	0.72	0.73	0.71	0.71	0.72	0.70
Year	2020	2025	2030	2035	2040	2045	2050			
Pig iron/Crude steel	0.71	0.71	0.71	0.71	0.71	0.71	0.71			
Crude steel (Mt)	1815	1963	2066	2179	2298	2423	2554			
Pig iron (Mt)	1289	1394	1467	1547	1632	1720	1813			
BFS/Pig iron	0.260	0.250	0.240	0.230	0.220	0.210	0.200			
BFS (Mt)	335	348	352	356	359	361	363			
SS/Crude steel	0.130	0.125	0.120	0.115	0.110	0.105	0.100			
SS (Mt)	236	245	248	251	253	254	255			

Supplementary Table 4-Chemical compositions and heat capacities of BFS, SS and fuel

Chemical compositions of BFS, SS and fuel

Slag	Composition	CaO	SiO_2	MgO	Al_2O_3	Fe_2O_3	FeO	Basicity*	Fuel (Wt. %)	
BFS	Wt. %	41.90%	38.10%	12.00%	8.00%			1.1		
	Mole %	42.48%	36.04%	17.03%	4.45%				C	78.00%
SS	Wt. %	42.86%	17.14%	5.00%	10.00%	8.75%	16.25%	2.5	H	2.00%
	Mole %	49.23%	18.38%	8.04%	6.31%	3.52%	14.52%		O	20.00%

Heat capacities of BFS and SS (kJ/kg/K)

Slag	T/^{o}C	100	300	500	700	900	1100	1300	1500	1700
BFS	Summation of oxides	0.97	1.06	1.11	1.14	1.15	1.16	1.17	1.18	1.18
	Direct calculation	0.85	0.98	1.05	1.08	1.12	1.14	1.18	1.21	1.22
SS	Summation of oxides	0.90	0.97	1.01	1.03	1.04	1.06	1.07	1.08	1.08
	Direct calculation	0.80	0.92	0.97	1.02	1.04	1.15	1.16	1.17	1.17

*Defined as the mass ratio of CaO to SiO_2, CaO/SiO_2

Supplementary Table 5-Important parameters for the cost-benefit analysis

Resource	Price	Unit
Coal	70.00	US$/tonne
Natural gas	6.00	US$/MMBtu
CO_2 price	30.00	US$/tonne of CO_2
Glassy BFS	75.00	US$/tonne
Crystalline SS	5.88	US$/tonne
Electricity	0.15	US$/kwh
Emission capacity	210.00	Pounds CO_2/MMBtu
Weight transfer	0.45	kg/pound
Energy transfer	1.06E+09	J/MMBtu

Supplementary Table 6-Cost-benefit analysis of Scheme 1 per tonne of slag

BFS			SS		
Cost (US$/tonne of slag)	Capital	7.83	Cost (US$/tonne of slag)	Capital	7.83
	Labour	0.77		Labour	0.77
	Maintenance	0.73		Maintenance	0.73
	Energy	1.20		Energy	1.20
	Total	10.52		Total	10.52
Benefit (US$/tonne of slag)	Slag	75.00	Benefit (US$/tonne of slag)	Slag	5.88
	Steam	7.36		Steam	9.50
	CO_2 avoided	2.80		CO_2 avoided	3.62
	Total	85.16		Total	19.00
Net revenue (US$/tonne of slag)		74.64	Net revenue (US$/tonne of slag)		8.48

Supplementary Table 7-Cost-benefit analysis of Scheme 2 per tonne of slag

BFS			SS		
Cost (US$/tonne of slag)	Capital	7.83	Cost (US$/tonne of slag)	Capital	7.83
	Labour	0.77		Labour	0.77
	Maintenance	0.73		Maintenance	0.73
	Energy	1.44		Energy	1.44
	Resource-Coal	0.97		Resource-Coal	1.39
	Resource-Waste	0.00		Resource-Waste	0.00
	CO_2 cost-Coal	1.19		CO_2 cost-Coal	1.71
	CO_2 cost-Waste	0.00		CO_2 cost-Waste	0.00
	Total-Coal	12.93		Total-Coal	13.87
	Total-Waste	10.76		Total-Waste	10.76
Benefit (US$/tonne of slag)	Slag	75.00	Benefit (US$/tonne of slag)	Slag	5.88
	Syngas	5.97		Syngas	5.94
	Steam	3.93		Steam	5.64
	CO_2 avoided-Coal	3.76		CO_2 avoided-Coal	4.39
	CO_2 avoided-Waste	3.77		CO_2 avoided-Waste	4.41
	Total-Coal	88.66		Total-Coal	21.85
	Total-Waste	88.67		Total-Waste	21.86
Net revenue (US$/tonne of slag)-Coal		75.73	Net revenue (US$/tonne of slag)-Coal		7.98
Net revenue (US$/tonne of slag)-Waste		77.90	Net revenue (US$/tonne of slag)-Waste		11.10

Supplementary Table 8-Cost-benefit analysis of Scheme 3 per tonne of slag

BFS			SS		
Cost (US$/tonne of slag)	Capital	7.83	Cost (US$/tonne of slag)	Capital	7.83
	Labour	0.77		Labour	0.77
	Maintenance	0.73		Maintenance	0.73
	Energy	1.44		Energy	1.44
	Resource-Coal	1.09		Resource-Coal	1.56
	Resource-Waste	0.00		Resource-Waste	0.00
	CO_2 cost-Coal	1.33		CO_2 cost-Coal	1.91
	CO_2 cost-Waste	0.00		CO_2 cost-Waste	0.00
	Total-Coal	13.19		Total-Coal	14.24
	Total-Waste	10.76		Total-Waste	10.76
Benefit (US$/tonne of slag)	Slag	75.00	Benefit (US$/tonne of slag)	Slag	5.88
	Syngas	5.49		Syngas	5.24
	Steam	4.37		Steam	6.27
	CO_2 avoided-Coal	3.74		CO_2 avoided-Coal	4.37
	CO_2 avoided-Waste	3.75		CO_2 avoided-Waste	4.39
	Total-Coal	88.60		Total-Coal	21.76
	Total-Waste	88.61		Total-Waste	21.78
Net revenue (US$/tonne of slag)-Coal		75.41	Net revenue (US$/tonne of slag)-Coal		7.52
Net revenue (US$/tonne of slag)-Waste		77.84	Net revenue (US$/tonne of slag)-Waste		11.01

Supplementary Table 9-Cost-benefit analysis of Scheme 4 per tonne of slag

BFS			SS		
Cost (US$/tonne of slag)	Capital	7.83	Cost (US$/tonne of slag)	Capital	7.83
	Labour	0.77		Labour	0.77
	Maintenance	0.73		Maintenance	0.73
	Energy	1.44		Energy	1.44
	Resource-Coal	0.99		Resource-Coal	1.42
	Resource-Waste	0.00		Resource-Waste	0.00
	CO_2 cost-Coal	1.22		CO_2 cost-Coal	1.74
	CO_2 cost-Waste	0.00		CO_2 cost-Waste	0.00
	Total-Coal	12.97		Total-Coal	13.93
	Total-Waste	10.76		Total-Waste	10.76
Benefit (US$/tonne of slag)	Slag	75.00	Benefit (US$/tonne of slag)	Slag	5.88
	Syngas	5.91		Syngas	5.85
	Steam	4.01		Steam	5.75
	CO_2 avoided-Coal	3.77		CO_2 avoided-Coal	4.40
	CO_2 avoided-Waste	3.78		CO_2 avoided-Waste	4.42
	Total-Coal	88.68		Total-Coal	21.88
	Total-Waste	88.69		Total-Waste	21.90
Net revenue (US$/tonne of slag)-Coal		75.71	Net revenue (US$/tonne of slag)-Coal		7.95
Net revenue (US$/tonne of slag)-Waste		77.93	Net revenue (US$/tonne of slag)-Waste		11.13

Supplementary Table 10-Cost-benefit analysis of Scheme 5 per tonne of slag

BFS			SS		
Cost (US$/tonne of slag)	Capital	7.83	Cost (US$/tonne of slag)	Capital	7.83
	Labour	0.77		Labour	0.77
	Maintenance	0.73		Maintenance	0.73
	Energy	1.44		Energy	1.44
	Resource-Coal	0.97		Resource-Coal	1.39
	Resource-Waste	0.00		Resource-Waste	0.00
	CO_2 cost-Coal	1.19		CO_2 cost-Coal	1.70
	CO_2 cost-Waste	0.00		CO_2 cost-Waste	0.00
	Total-Coal	12.92		Total-Coal	13.86
	Total-Waste	10.76		Total-Waste	10.76
Benefit (US$/tonne of slag)	Slag	75.00	Benefit (US$/tonne of slag)	Slag	5.88
	Syngas	5.99		Syngas	5.97
	Steam	3.90		Steam	5.60
	CO_2 avoided-Coal	3.76		CO_2 avoided-Coal	4.39
	CO_2 avoided-Waste	3.77		CO_2 avoided-Waste	4.41
	Total-Coal	88.65		Total-Coal	21.84
	Total-Waste	88.66		Total-Waste	21.85
Net revenue (US$/tonne of slag)-Coal		75.73	Net revenue (US$/tonne of slag)-Coal		7.98
Net revenue (US$/tonne of slag)-Waste		77.90	Net revenue (US$/tonne of slag)-Waste		11.09

Supplementary Table 11-Summary of the economics of various schemes per tonne of crude steel

<table>
<tr><th>Cost-Benefit</th><th>Scheme 1</th><th>Scheme 2</th><th>Scheme 3</th><th>Scheme 4</th><th>Scheme 5</th></tr>
<tr><td>Cost-Coal (US$/tonne of crude steel)</td><td rowspan="2">3.31</td><td>4.19</td><td>4.28</td><td>4.21</td><td>4.19</td></tr>
<tr><td>Cost-Waste (US$/tonne of crude steel)</td><td>3.39</td><td>3.39</td><td>3.39</td><td>3.39</td></tr>
<tr><td>Benefit-Coal (US$/tonne of crude steel)</td><td rowspan="2">18.19</td><td>19.21</td><td>19.18</td><td>19.21</td><td>19.20</td></tr>
<tr><td>Benefit-Waste (US$/tonne of crude steel)</td><td>19.21</td><td>19.19</td><td>19.22</td><td>19.21</td></tr>
<tr><td>Net revenue-Coal (US$/tonne of crude steel)</td><td rowspan="2">14.88</td><td>15.02</td><td>14.90</td><td>15.01</td><td>15.02</td></tr>
<tr><td>Net revenue-Waste (US$/tonne of crude steel)</td><td>15.82</td><td>15.80</td><td>15.83</td><td>15.82</td></tr>
</table>

Supplementary Table 12-Summary of the economics of various schemes for a steel plant with an annual crude steel output of 1 Mt (US$, million)

Scheme	Scheme 1	Scheme 2	Scheme 3	Scheme 4	Scheme 5
Fuel-Coal	14.88	14.90	15.02	15.02	15.01
Fuel-Waste		15.80	15.82	15.82	15.83

Supplementary Table 13-Capital cost of the granulation process in Schemes 1-5

Capital cost type	Cost for a plant producing 300000 t BFS annually (US$)
Granulator	780500
Heat exchanger & boiler	490000
Offgas handing system	127050
Feed and product handling system	127050
Total equipment cost	1524600
Total installed equipment cost	1829520
Instrumentation & controls	152460
EPCM (Engineering, procurement, construction, management)	365904
Total capital cost	2347884
Capital cost per tonne of slag	7.83

Supplementary Table 14-Results for CO_2 gasification

CO_2/mol	H_2 content in syngas	CO content in syngas	H_2 content in dry syngas	CO content in dry syngas	CE	HE
0.5	11.39%	78.44%	11.53%	79.38%	95.84%	90.70%
1.0	6.56%	82.77%	6.61%	83.37%	175.41%	90.20%
1.5	4.41%	69.59%	4.48%	70.59%	184.76%	75.69%
2.0	3.16%	59.12%	3.22%	60.17%	186.52%	64.36%
2.5	2.37%	51.39%	2.42%	52.36%	187.82%	55.99%
3.0	1.85%	45.44%	1.88%	46.32%	188.82%	49.55%
3.5	1.48%	40.73%	1.51%	41.50%	189.61%	44.44%
4.0	1.21%	36.91%	1.23%	37.58%	190.26%	40.28%
4.5	1.01%	33.74%	1.03%	34.33%	190.79%	36.84%
5.0	0.85%	31.07%	0.87%	31.60%	191.24%	33.94%
5.5	0.73%	28.79%	0.74%	29.26%	191.62%	31.46%
6.0	0.64%	26.83%	0.65%	27.24%	191.95%	29.32%
6.5	0.56%	25.11%	0.56%	25.49%	192.24%	27.46%
7.0	0.49%	23.61%	0.50%	23.94%	192.50%	25.81%
7.5	0.44%	22.27%	0.44%	22.57%	192.72%	24.36%
8.0	0.39%	21.07%	0.40%	21.35%	192.93%	23.06%
8.5	0.35%	20.00%	0.36%	20.26%	193.11%	21.89%
9.0	0.32%	19.03%	0.32%	19.26%	193.27%	20.83%
9.5	0.29%	18.15%	0.29%	18.37%	193.42%	19.87%
10.0	0.26%	17.35%	0.27%	17.55%	193.56%	19.00%

Supplementary Table 15-Results for H_2O gasification

H_2O/mol	H_2 content in syngas	CO content in syngas	H_2 content in dry syngas	CO content in dry syngas	CE	HE
0.5	47.16%	45.90%	48.55%	47.25%	59.02%	399.38%
1.0	48.18%	41.73%	50.65%	43.86%	90.13%	678.20%
1.5	46.39%	27.36%	55.14%	32.52%	72.77%	796.51%
2.0	42.95%	19.23%	57.52%	25.75%	60.74%	875.11%
2.5	39.48%	14.23%	59.06%	21.29%	52.06%	931.47%
3.0	36.32%	10.95%	60.14%	18.13%	45.52%	973.85%
3.5	33.52%	8.68%	60.94%	15.78%	40.43%	1006.78%
4.0	31.06%	7.05%	61.55%	13.97%	36.35%	1033.14%
4.5	28.90%	5.84%	62.04%	12.53%	33.02%	1054.63%
5.0	27.01%	4.91%	62.44%	11.36%	30.24%	1072.60%
5.5	25.33%	4.19%	62.77%	10.38%	27.89%	1087.74%
6.0	23.84%	3.62%	63.05%	9.56%	25.88%	1100.75%
6.5	22.51%	3.15%	63.28%	8.86%	24.14%	1111.97%
7.0	21.32%	2.77%	63.49%	8.26%	22.62%	1121.77%
7.5	20.24%	2.46%	63.67%	7.73%	21.28%	1130.47%
8.0	19.27%	2.19%	63.82%	7.27%	20.09%	1138.15%
8.5	18.38%	1.97%	63.96%	6.85%	19.02%	1145.05%
9.0	17.57%	1.78%	64.08%	6.49%	18.06%	1151.17%
9.5	16.83%	1.61%	64.20%	6.16%	17.20%	1156.78%
10.0	16.14%	1.47%	64.30%	5.86%	16.41%	1161.88%

Supplementary Table 16-Results for CO_2/H_2O gasification

CO_2/mol	H_2O/mol	H_2 content in syngas	CO content in syngas	H_2 content in dry syngas	CO content in dry syngas	CE	HE
0.1	1.9	41.00%	21.18%	54.02%	27.91%	66.89%	835.45%
0.2	1.8	39.05%	23.13%	50.64%	30.00%	73.05%	795.69%
0.3	1.7	37.09%	25.09%	47.35%	32.04%	79.23%	755.86%
0.4	1.6	35.13%	27.06%	44.17%	34.02%	85.42%	715.95%
0.5	1.5	33.16%	29.03%	41.07%	35.95%	91.62%	675.95%
0.6	1.4	31.19%	31.00%	38.07%	37.83%	97.85%	635.82%
0.7	1.3	29.22%	32.98%	35.15%	39.67%	104.08%	595.62%
0.8	1.2	27.24%	34.96%	32.31%	41.46%	110.34%	555.32%
0.9	1.1	25.26%	36.95%	29.54%	43.21%	116.61%	514.94%
1.0	1.0	23.27%	38.95%	26.85%	44.92%	122.89%	474.44%
1.1	0.9	21.28%	40.94%	24.22%	46.60%	129.19%	433.86%
1.2	0.8	19.29%	42.95%	21.66%	48.23%	135.50%	393.18%
1.3	0.7	17.29%	44.95%	19.16%	49.83%	141.84%	352.40%
1.4	0.6	15.28%	46.96%	16.73%	51.40%	148.18%	311.53%
1.5	0.5	13.28%	48.98%	14.35%	52.93%	154.53%	270.56%
1.6	0.4	11.26%	51.00%	12.02%	54.44%	160.90%	229.50%
1.7	0.3	9.24%	53.02%	9.75%	55.91%	167.29%	188.35%
1.8	0.2	7.22%	55.05%	7.52%	57.36%	173.69%	147.11%
1.9	0.1	5.19%	57.08%	5.35%	58.78%	180.10%	105.78%

Supplementary Figures

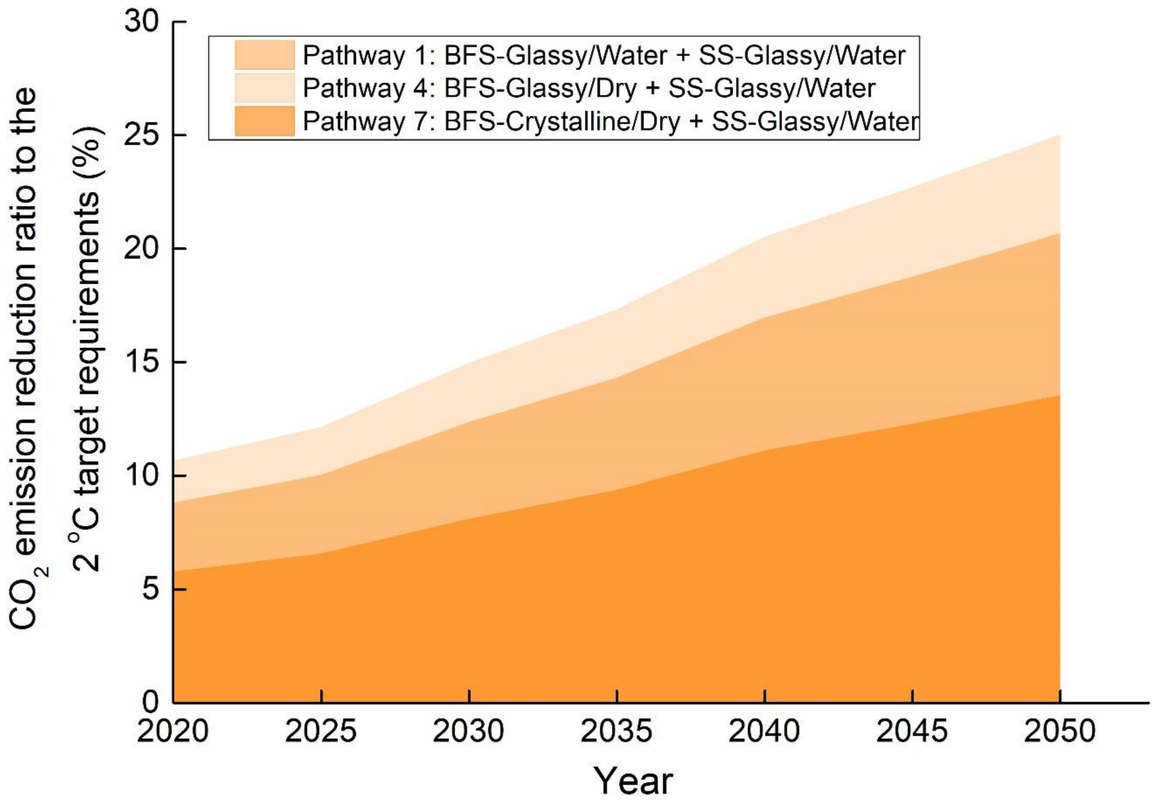

Supplementary Fig. 1-CO_2 emission reduction ratio to the 2 °C target requirements in the iron and steel sector by Pathways 1, 4 and 7.

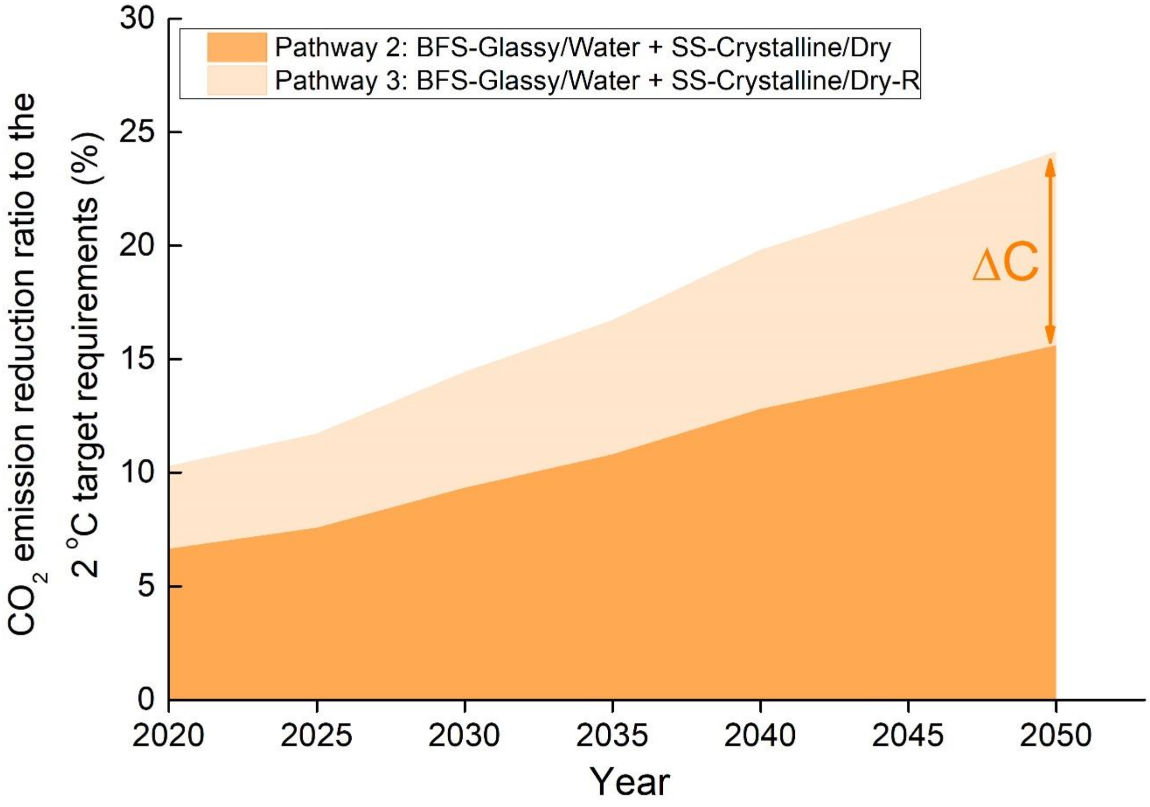

Supplementary Fig. 2-CO_2 emission reduction ratio to the 2 °C target requirements in the iron and steel sector by Pathways 2 and 3. The differences (ΔC) results from the further utilization of cooled crystalline SS, i.e., whether the high concentration of CaO in cooled crystalline SS could be recycled after necessary phase separations considering the technological advancements in the mid- to long term.

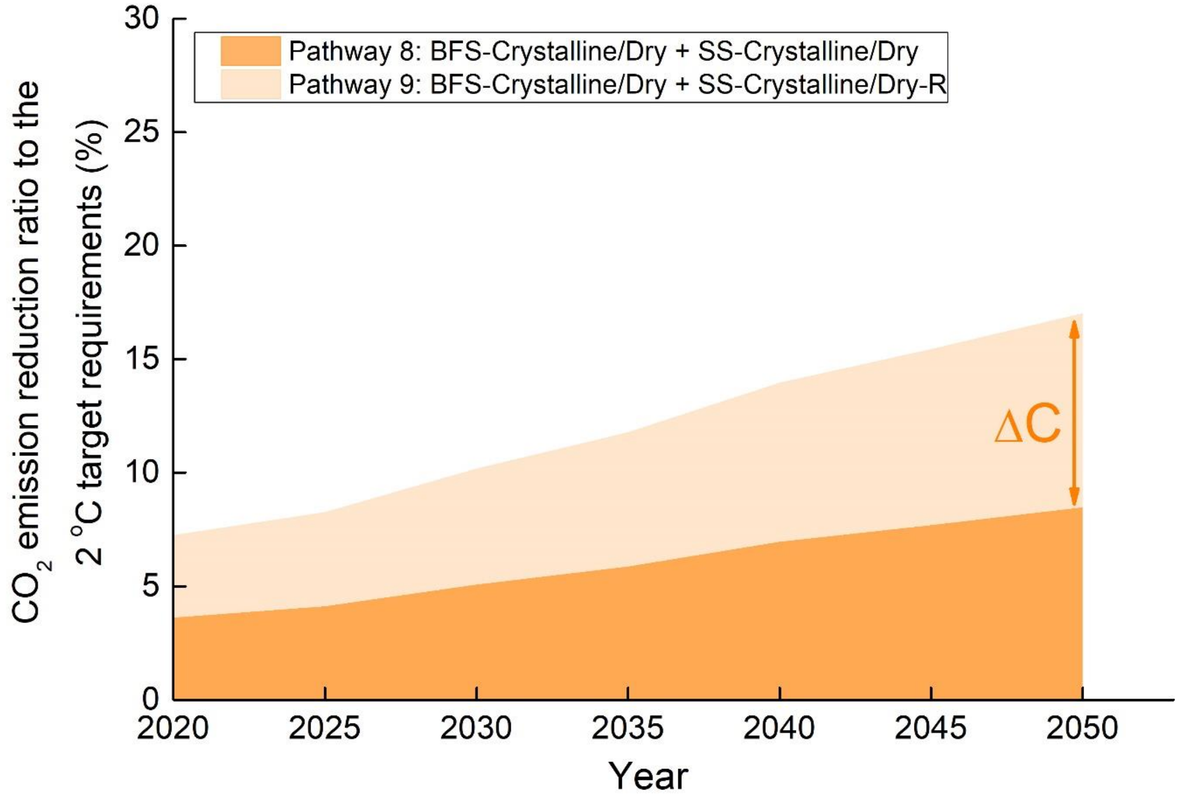

Supplementary Fig. 3-CO_2 emission reduction ratio to the 2 °C target requirements in the iron and steel sector by Pathways 8 and 9. The differences (ΔC) results from the further utilization of cooled crystalline SS, i.e., whether the high concentration of CaO in cooled crystalline SS could be recycled after necessary phase separations considering the technological advancements in the mid- to long term.

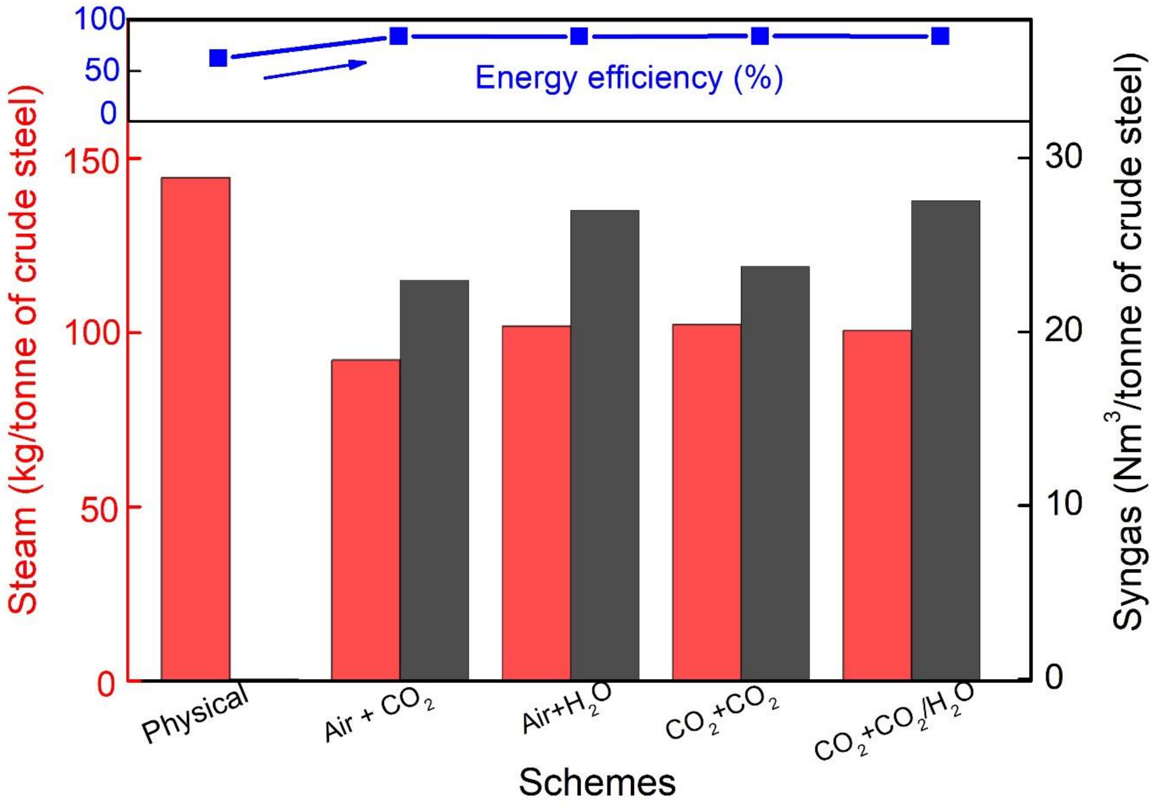

Supplementary Fig. 4-Yields of the main products, including syngas and steam, per tonne of crude steel in the different technological schemes and the energy efficiencies.

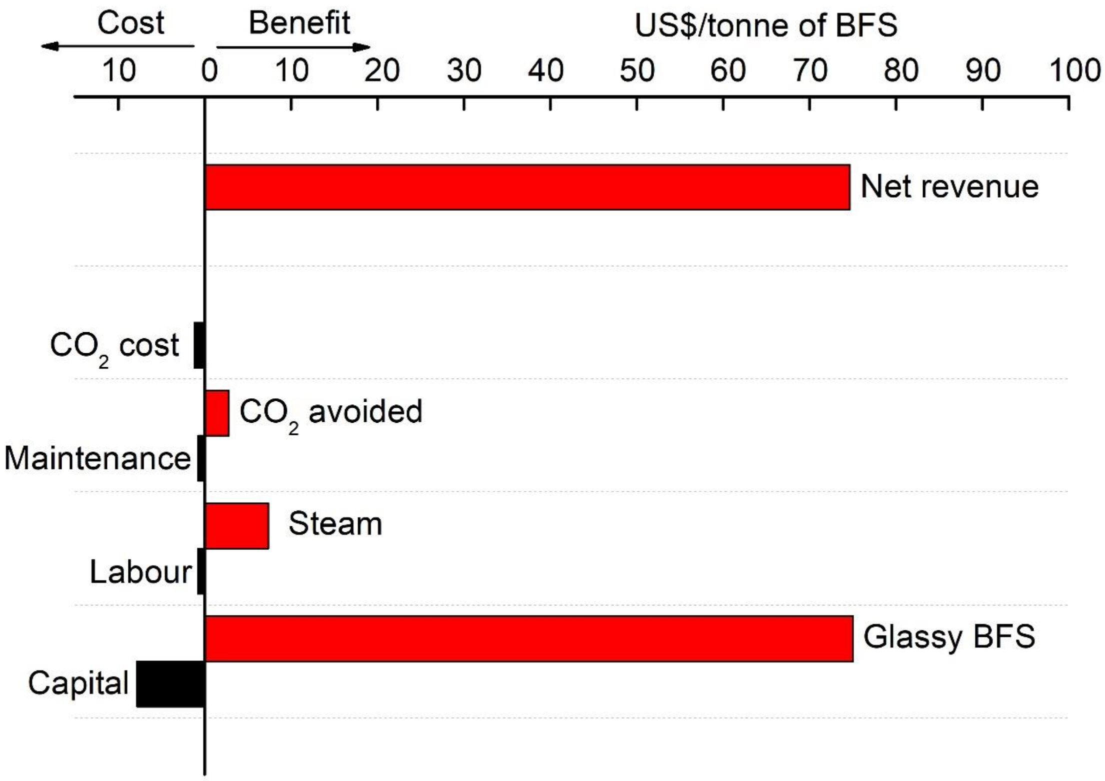

Supplementary Fig. 5-Cost-benefit analysis per tonne of glassy BFS for Scheme 1

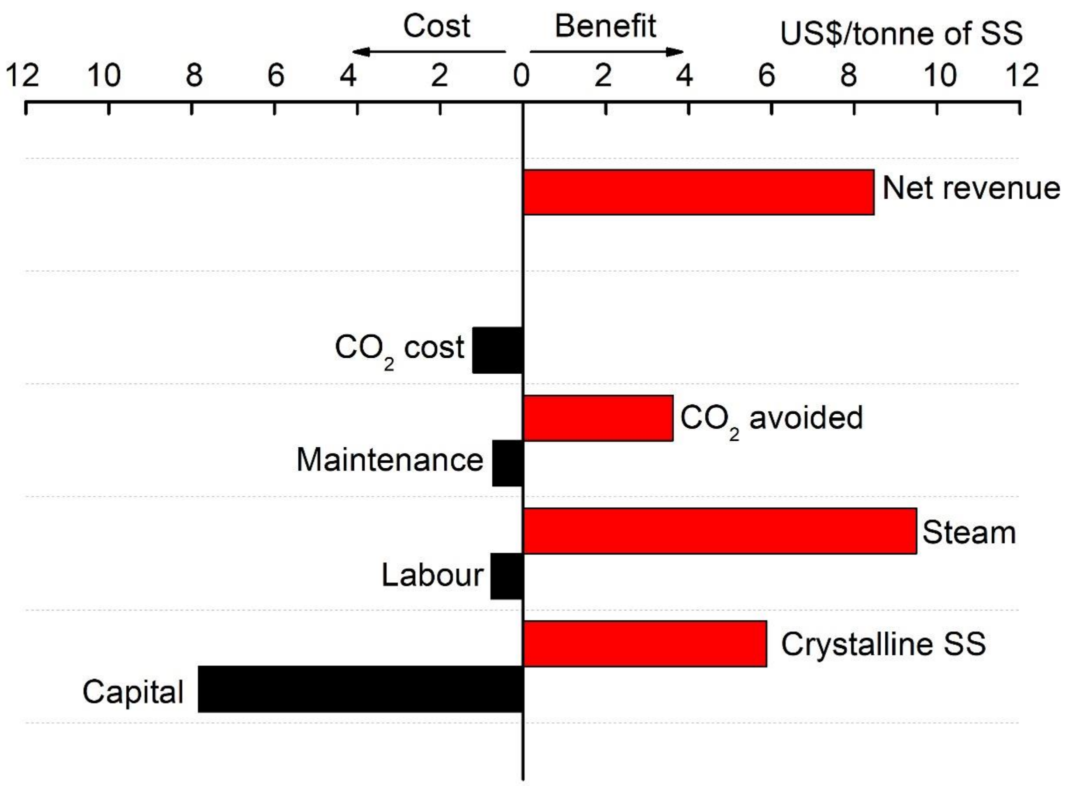

Supplementary Fig. 6-Cost-benefit analysis per tonne of crystalline SS for Scheme 1

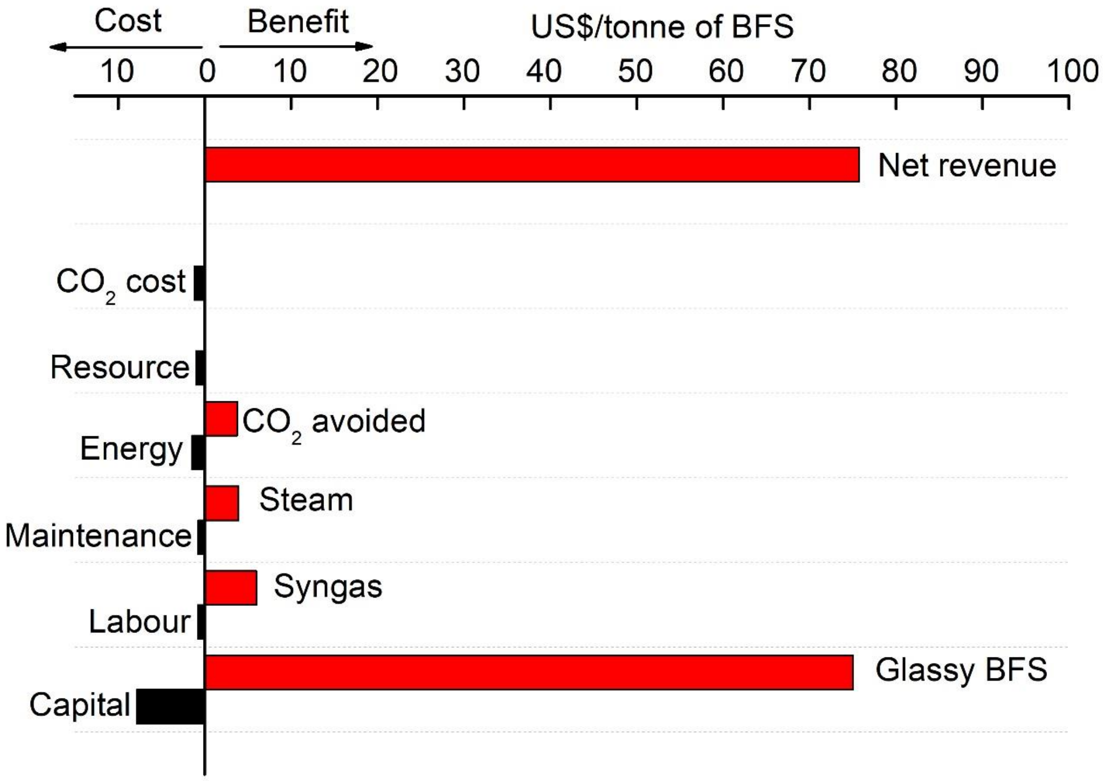

Supplementary Fig. 7-Cost-benefit analysis per tonne of glassy BFS for Scheme 2

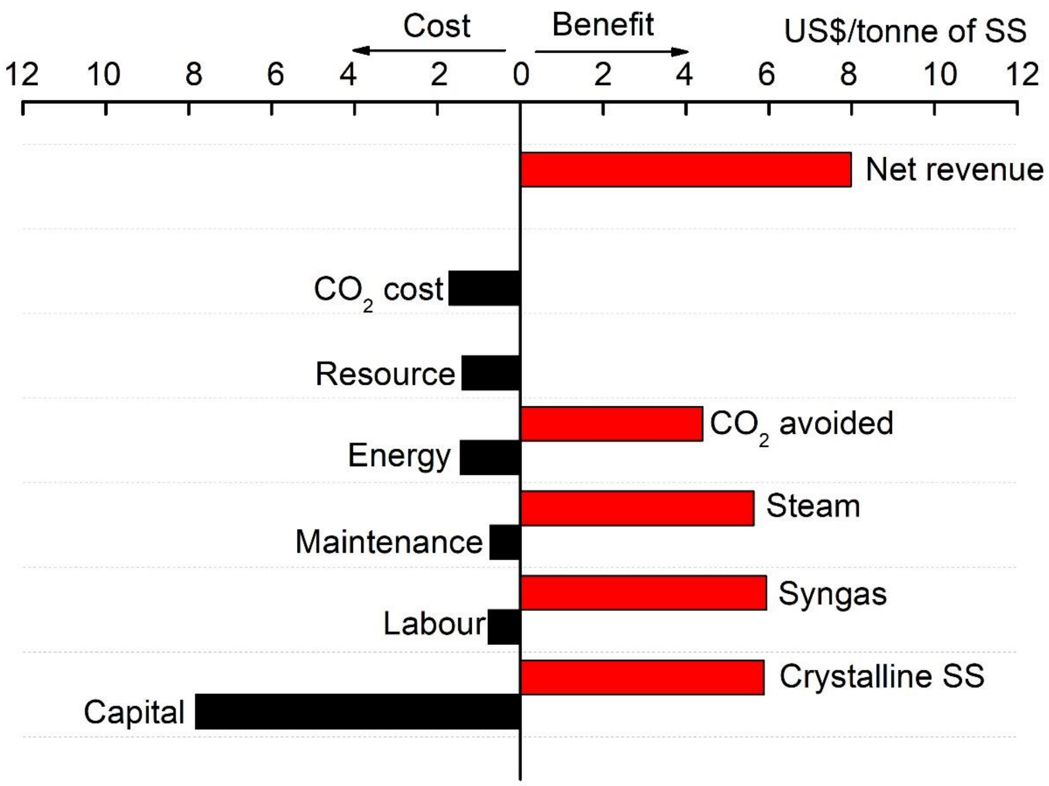

Supplementary Fig. 8-Cost-benefit analysis per tonne of crystalline SS for Scheme 2

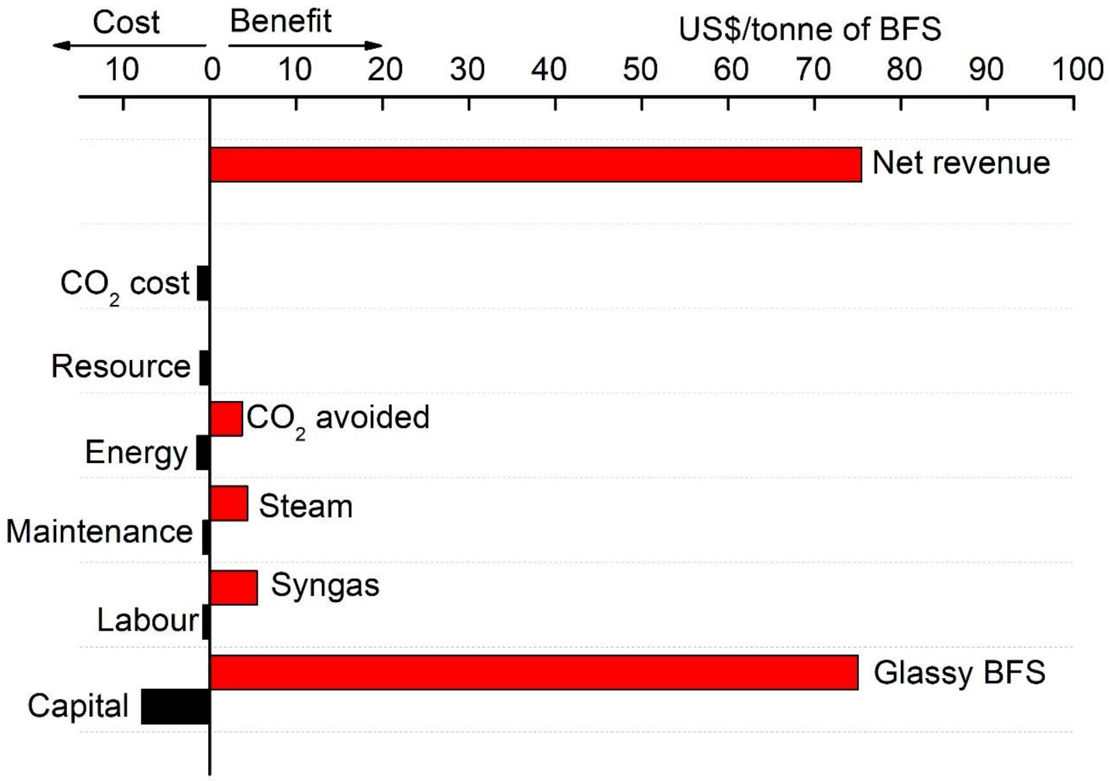

Supplementary Fig. 9-Cost-benefit analysis per tonne of glassy BFS for Scheme 3

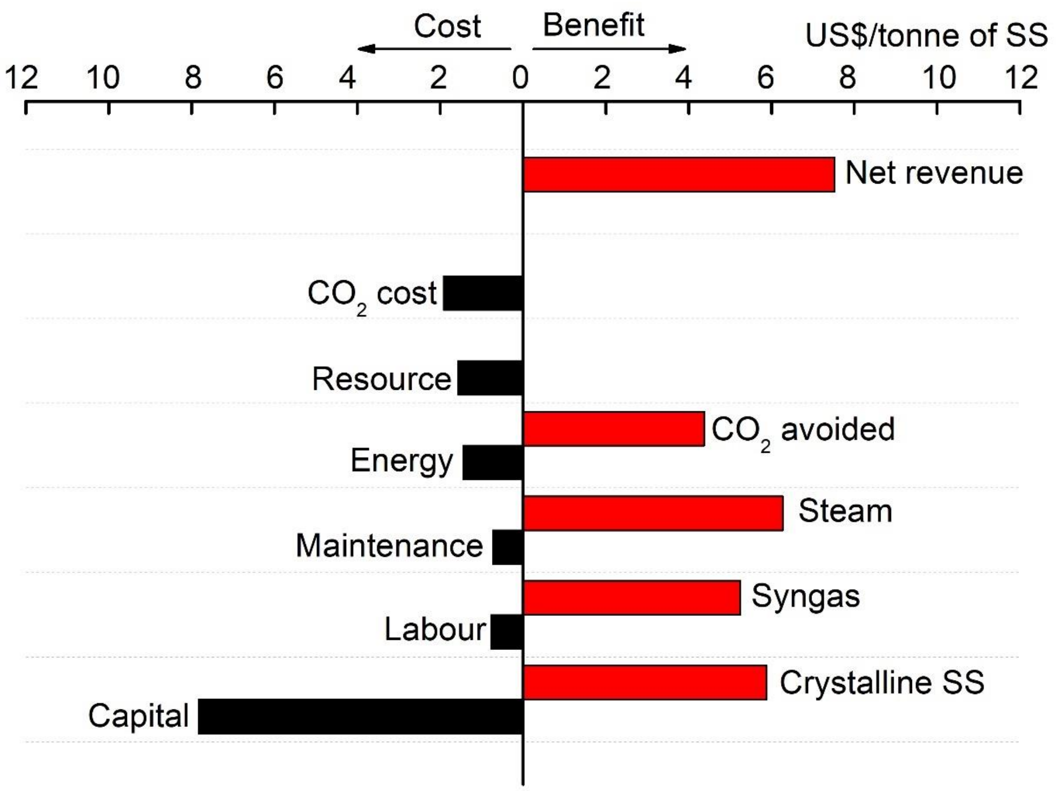

Supplementary Fig. 10-Cost-benefit analysis per tonne of crystalline SS for Scheme 3

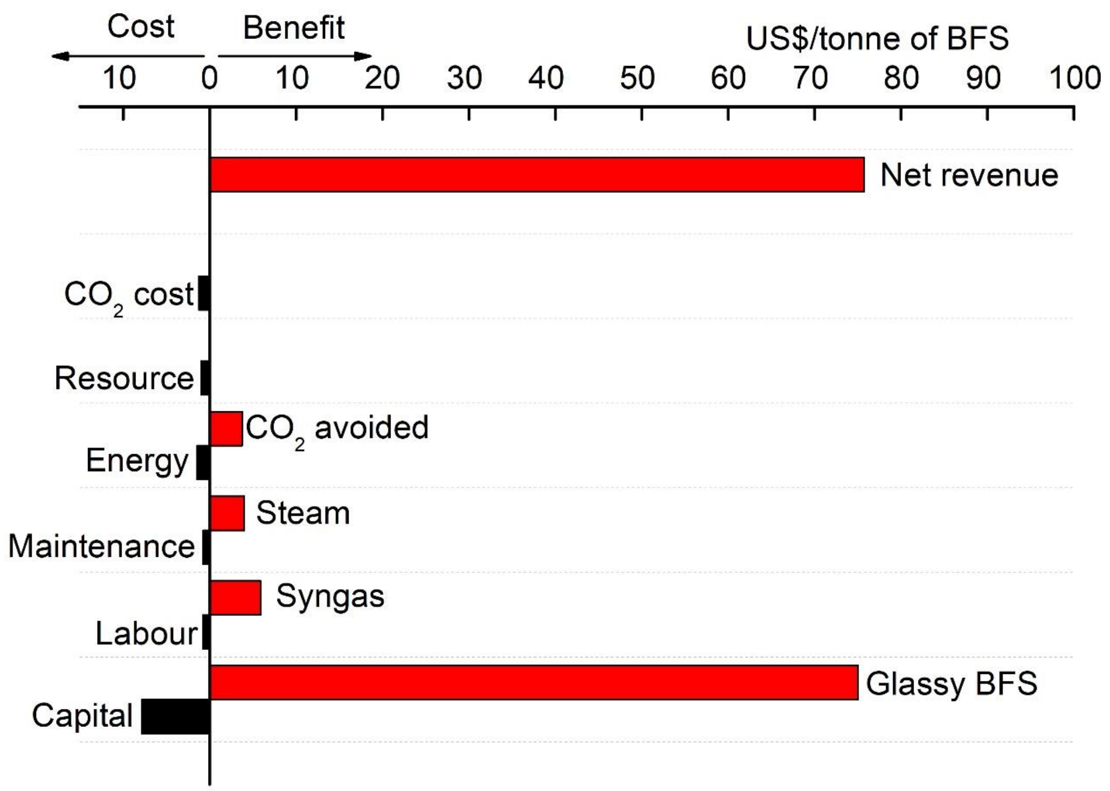

Supplementary Fig. 11-Cost-benefit analysis per tonne of glassy BFS for Scheme 4

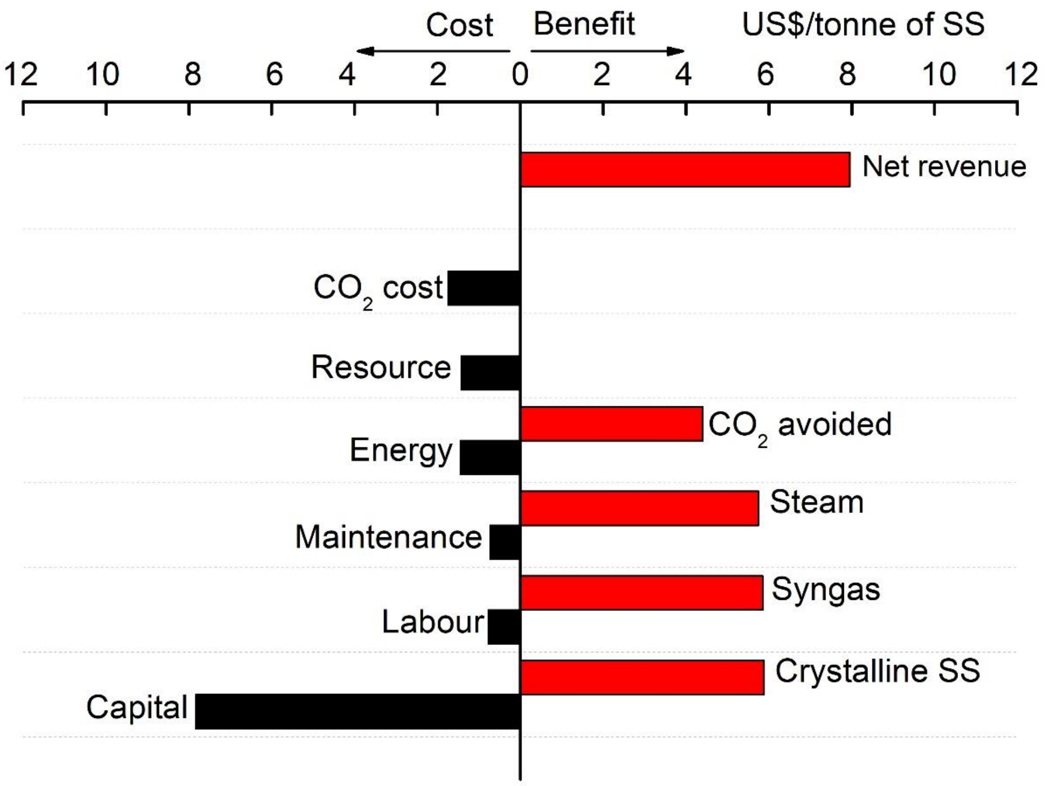

Supplementary Fig. 12-Cost-benefit analysis per tonne of crystalline SS for Scheme 4

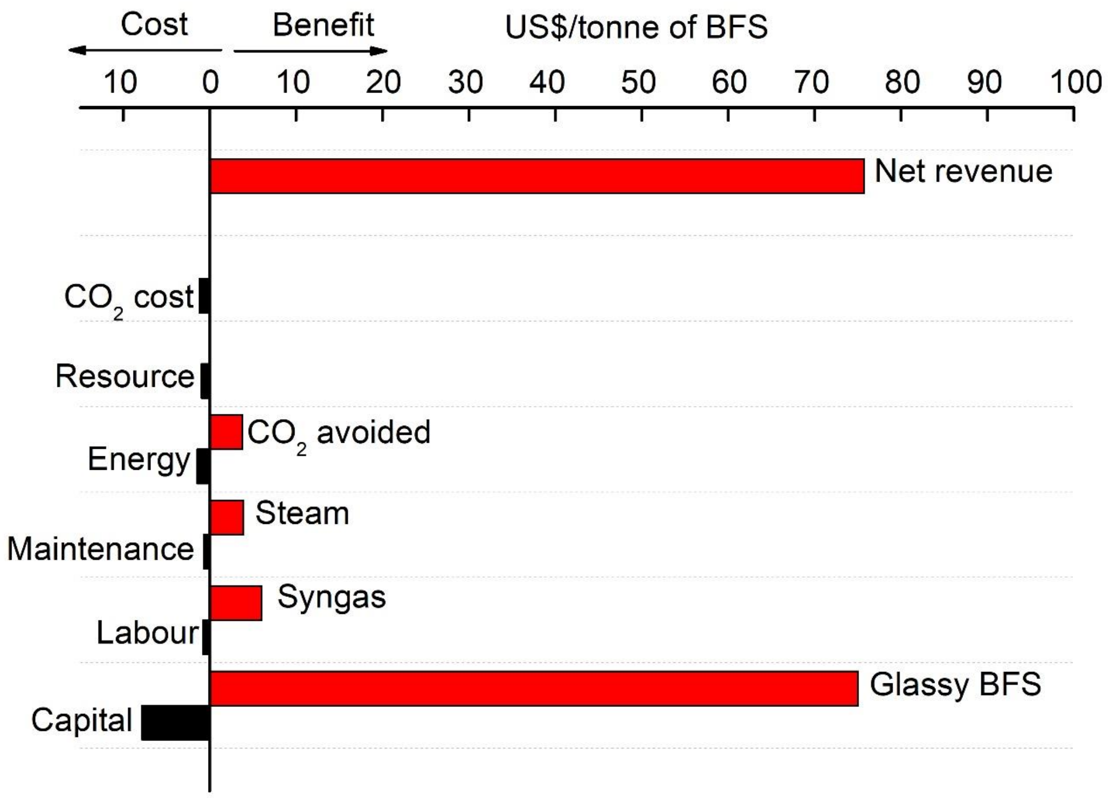

Supplementary Fig. 13-Cost-benefit analysis per tonne of glassy BFS for Scheme 5

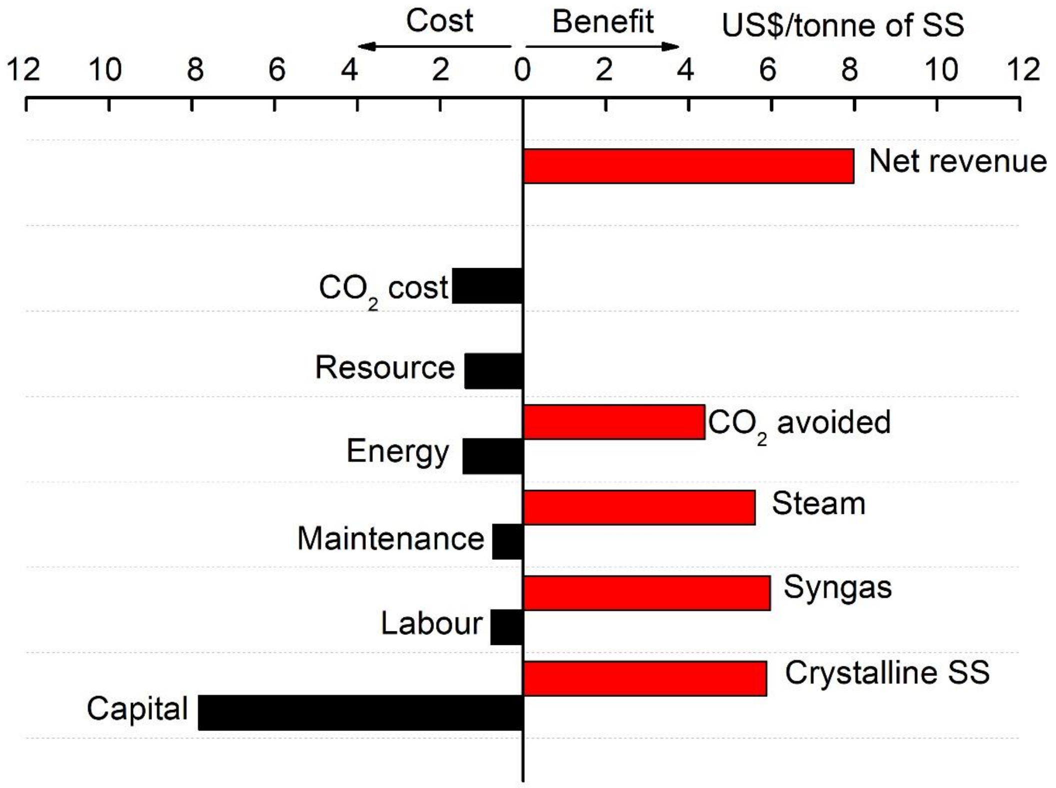

Supplementary Fig. 14-Cost-benefit analysis per tonne of crystalline SS for Scheme 5

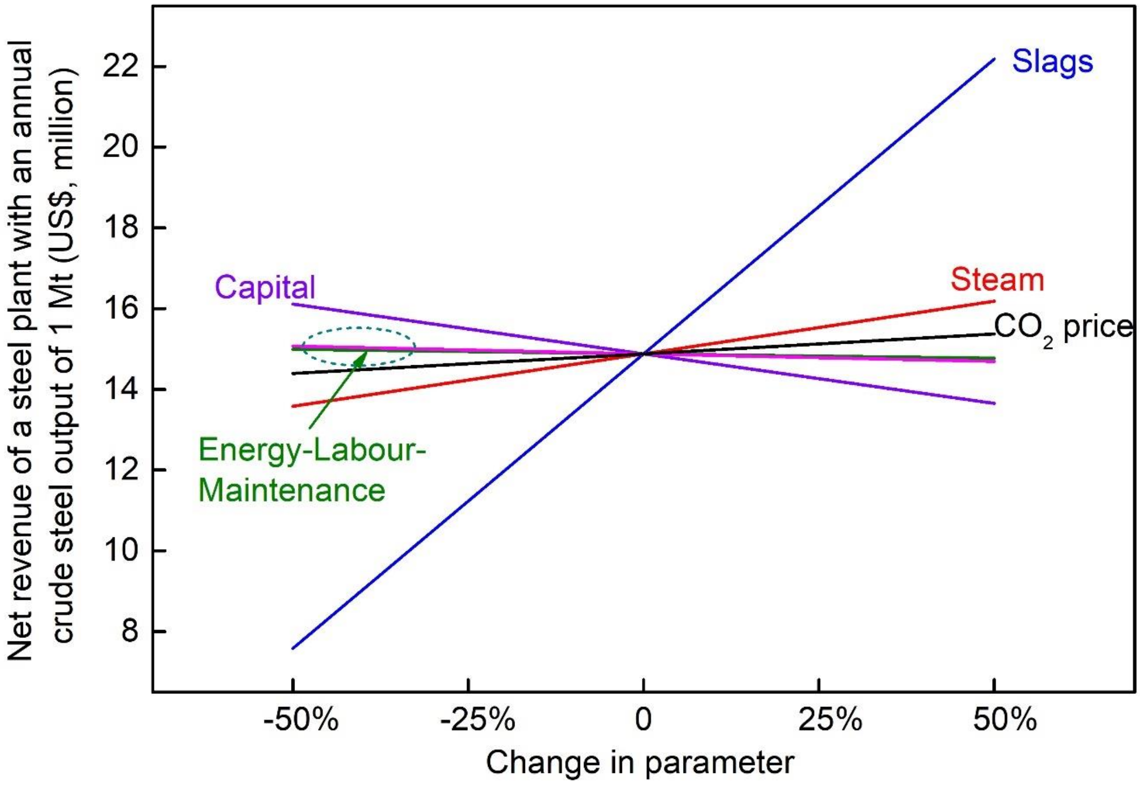

Supplementary Fig. 15-Economic sensitivity of a steel plant with an annual crude steel output of 1 Mt using Scheme 1

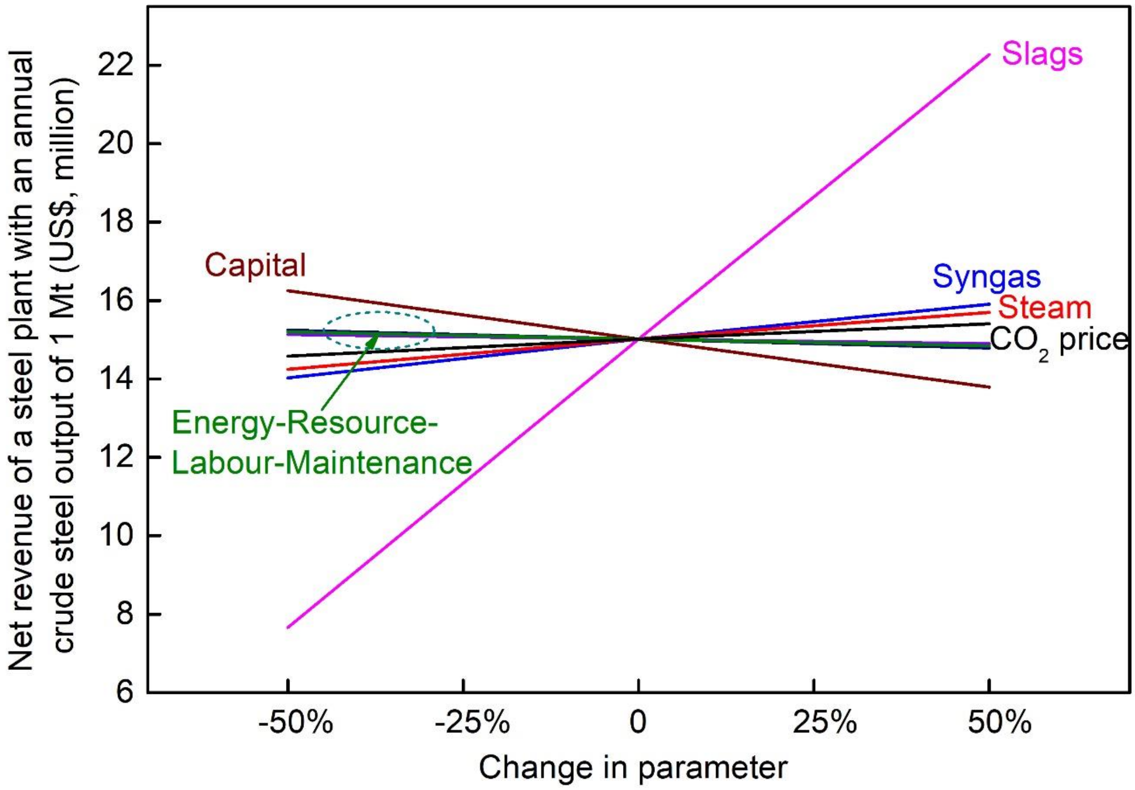

Supplementary Fig. 16-Economic sensitivity of a steel plant with an annual crude steel output of 1 Mt using Scheme 2

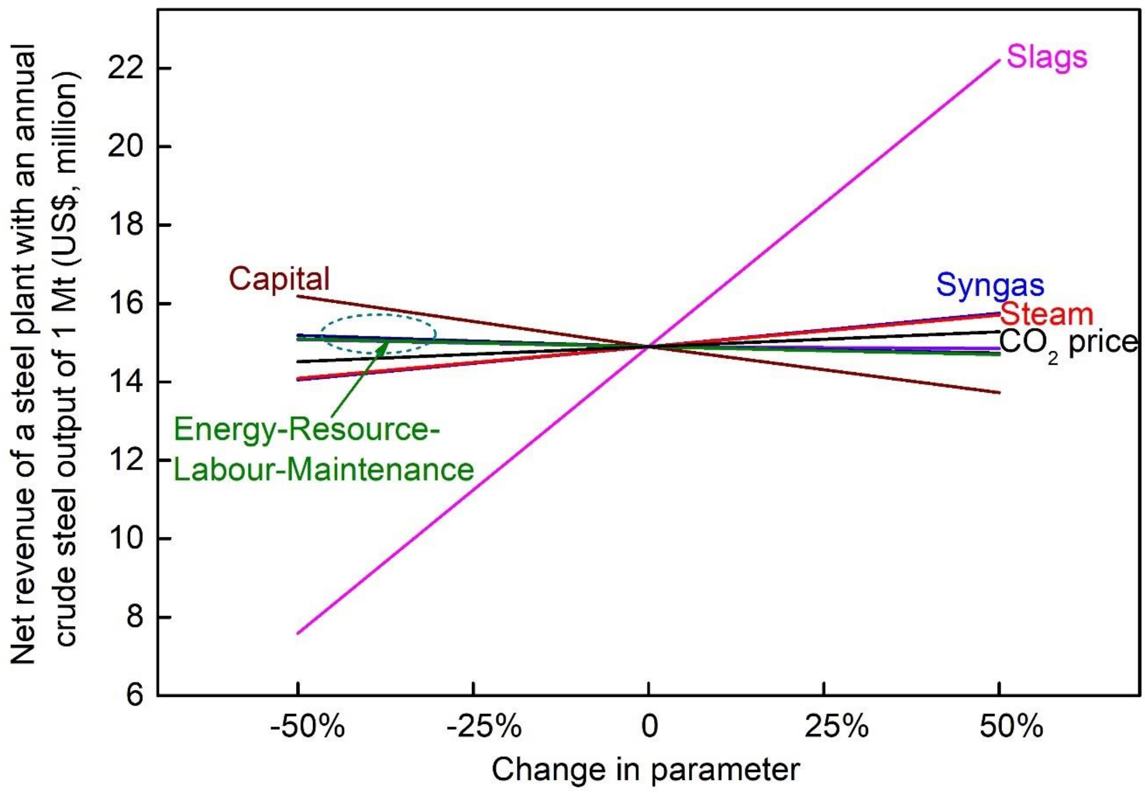

Supplementary Fig. 17-Economic sensitivity of a steel plant with an annual crude steel output of 1 Mt using Scheme 3

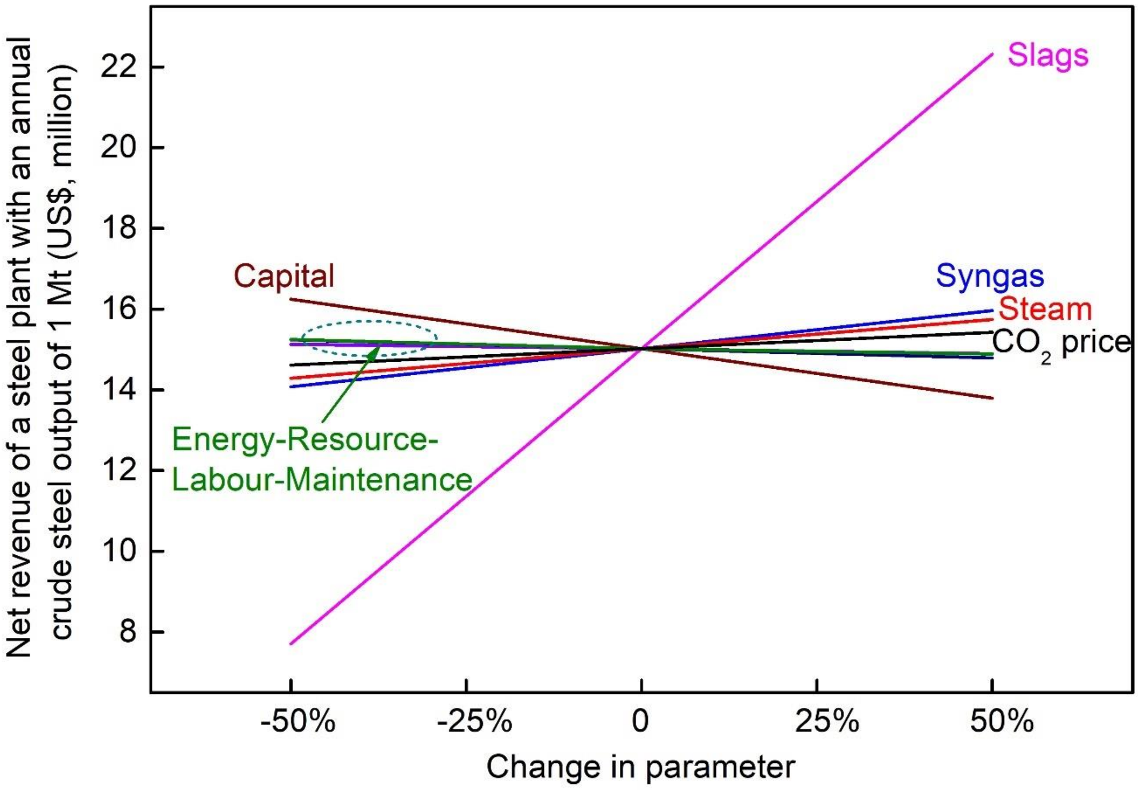

Supplementary Fig. 18-Economic sensitivity of a steel plant with an annual crude steel output of 1 Mt using Scheme 4

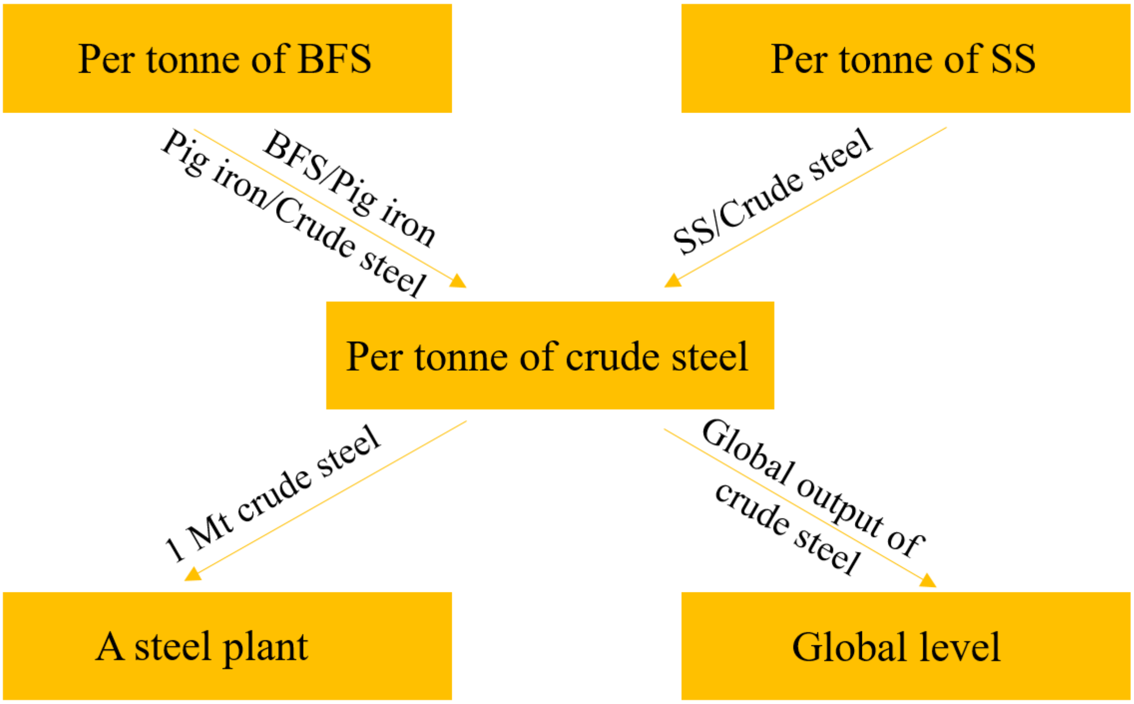

Supplementary Fig. 19-Research outline of the various pathways

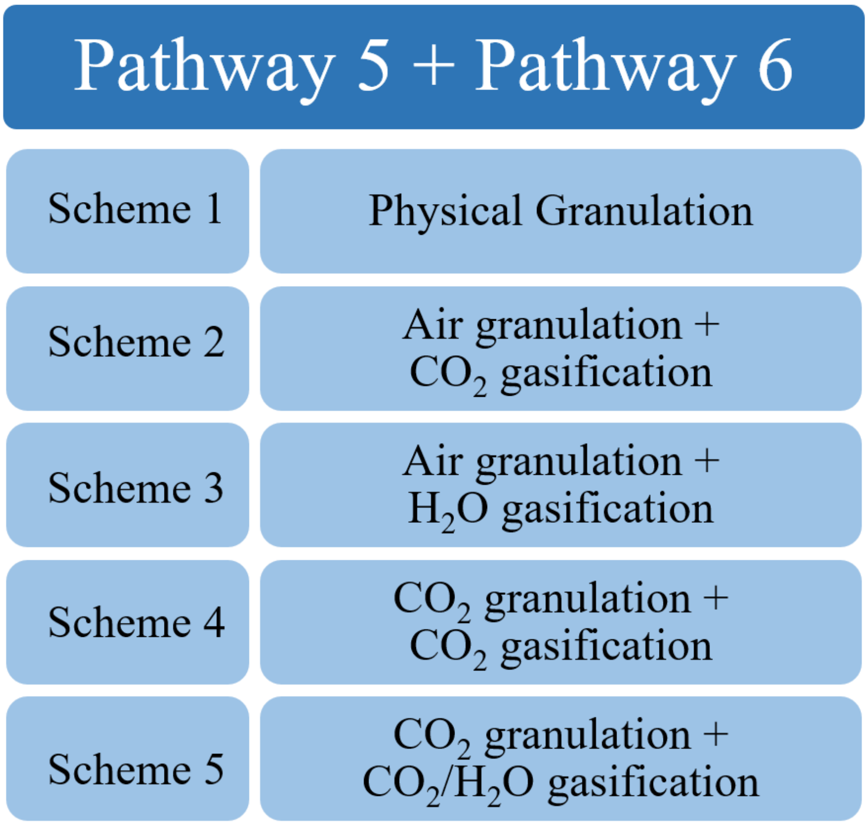

Supplementary Fig. 20-Five technology schemes to engineer Pathways 5 and 6

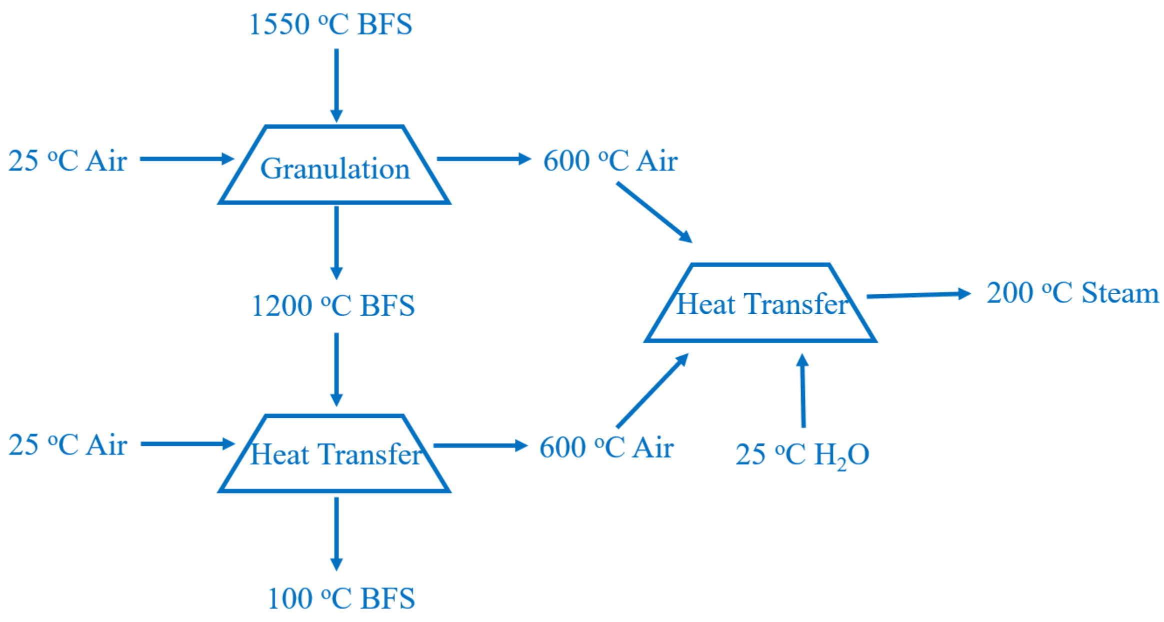

Supplementary Fig. 21-Technological process of Scheme 1

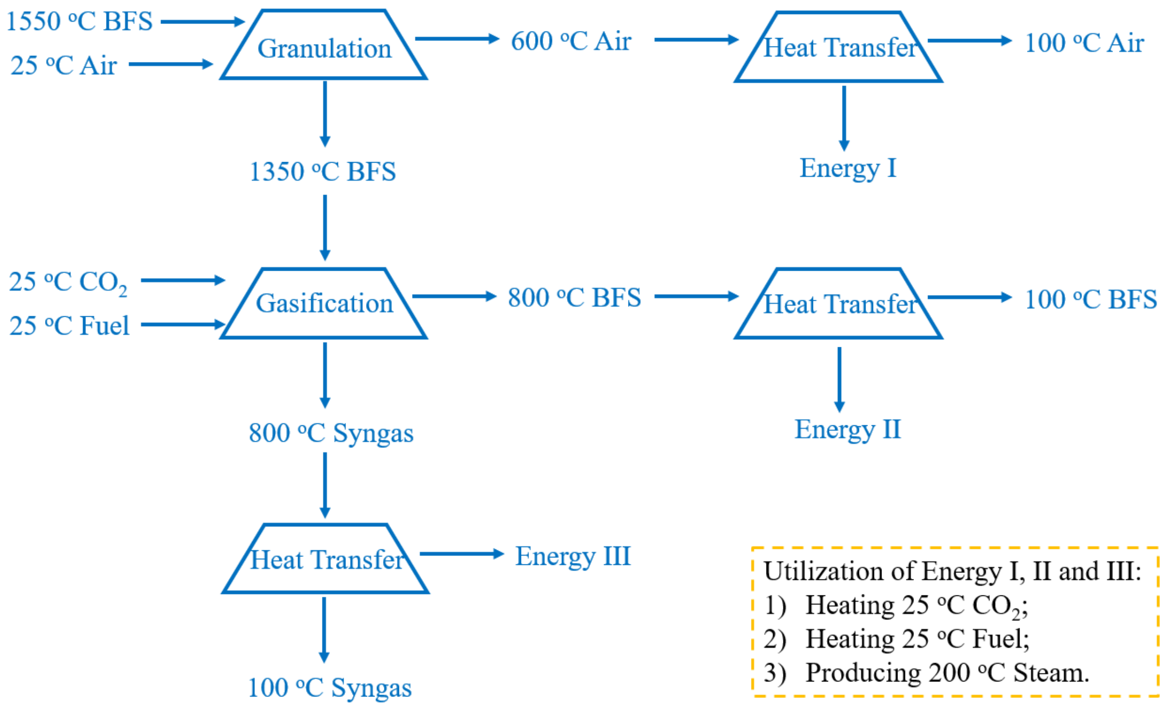

Supplementary Fig. 22-Technological process of Scheme 2

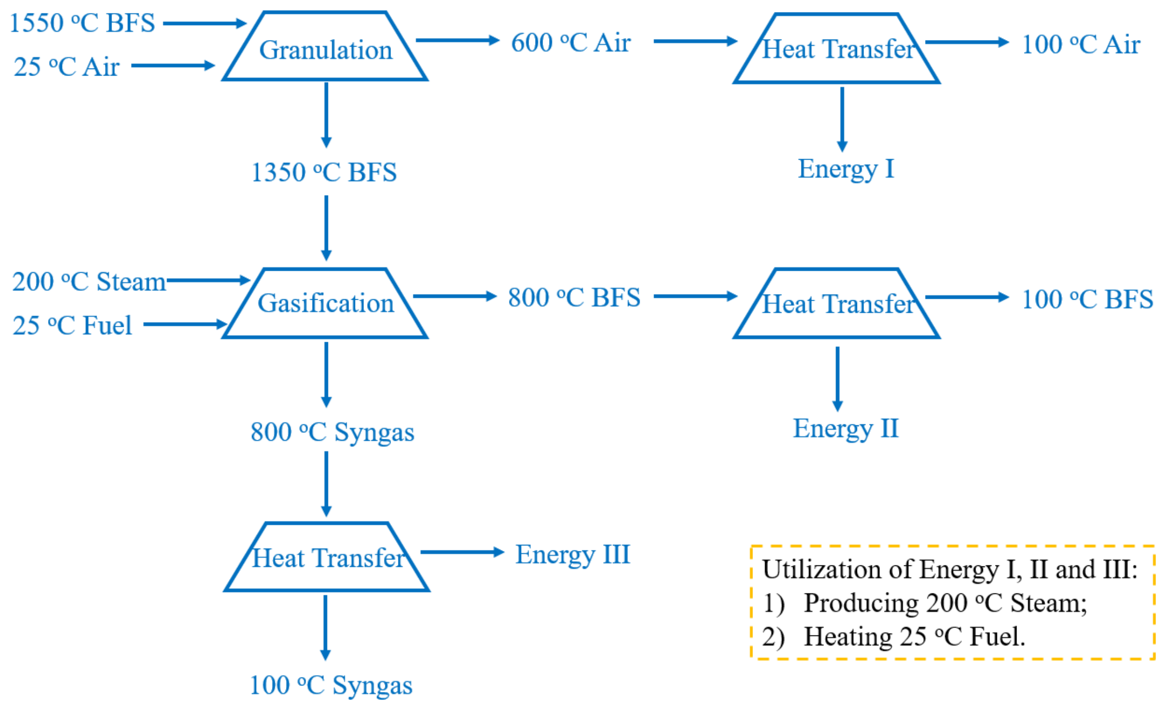

Supplementary Fig. 23-Technological process of Scheme 3

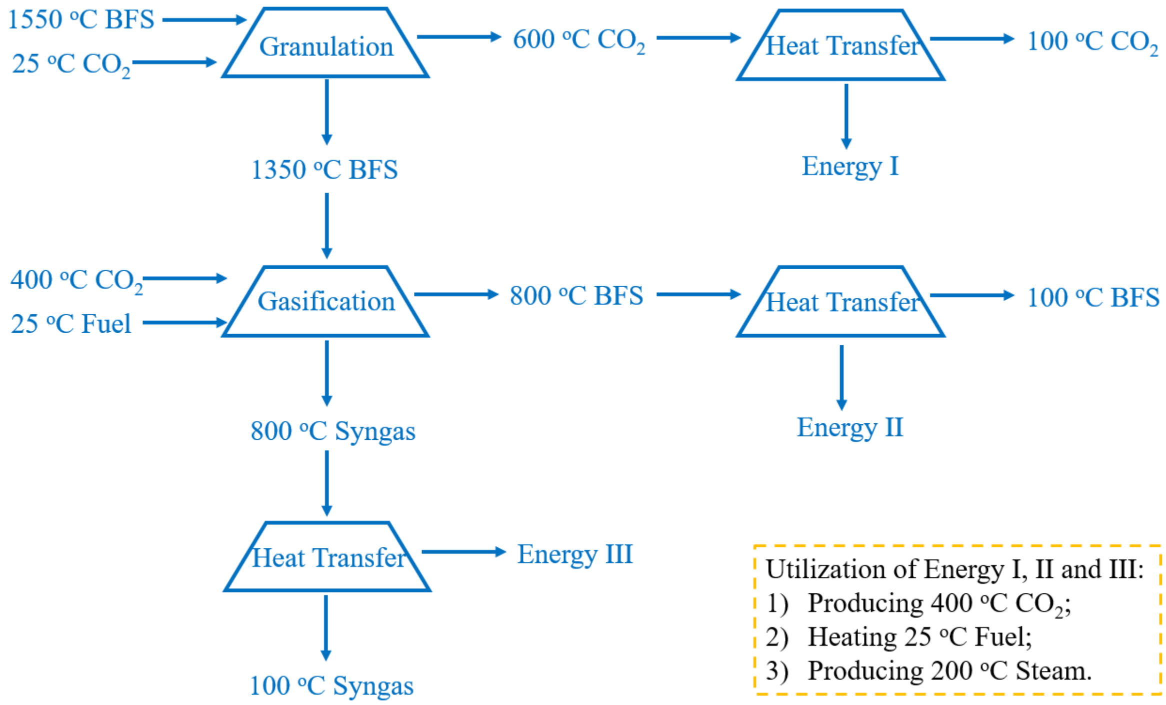

Supplementary Fig. 24-Technological process of Scheme 4

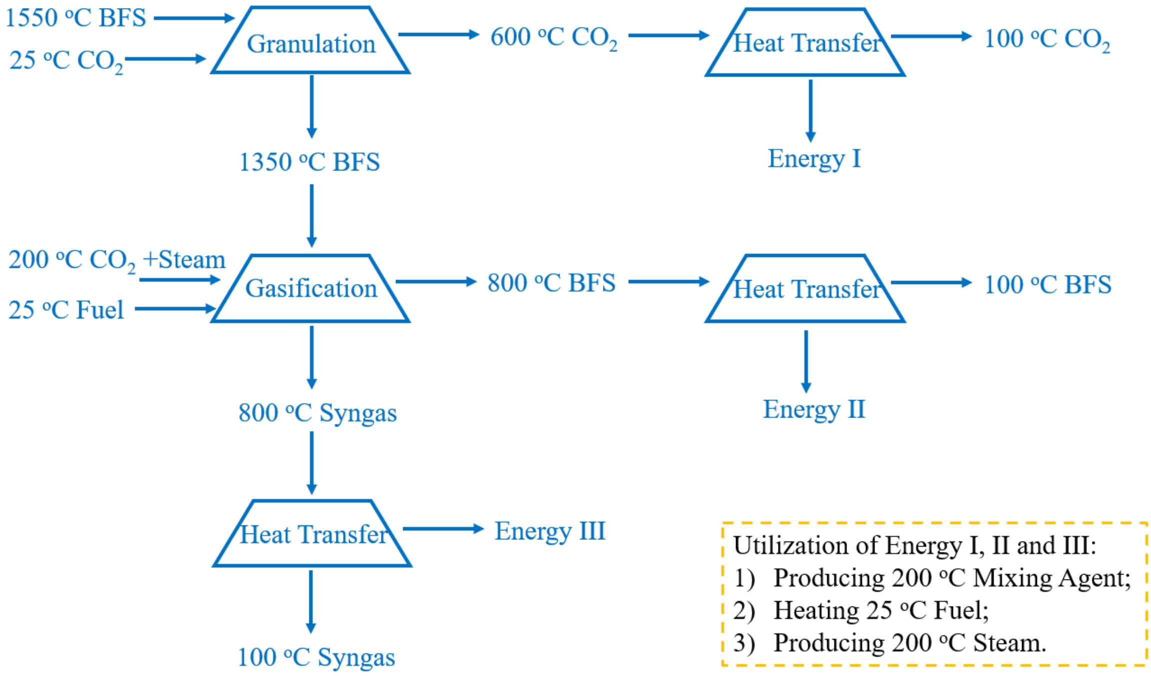

Supplementary Fig. 25-Technological process of Scheme 5

Supplementary References

1. Sun, Y. et al. Experimental investigation and modeling of cooling processes of high temperature slags. Energy 76, 761–767 (2014).

2. Sun, Y., Zhang, Z., Liu, L. & Wang, X. Multi-Stage control of waste heat recovery from high temperature slags based on time temperature transformation curves. Energies 7, 1673-1684 (2014).

3. Esfahani, S. & Barati, M. Effect of slag composition on the crystallization of synthetic CaO–SiO_2–Al_2O_3–MgO slags: Part I-Crystallization behavior. J. Non-Cryst. Solids 436, 35-43 (2016).

4. Esfahani, S., Mostaghel, S. & Barati, M. Effect of slag composition on the crystallization of synthetic CaO-SiO_2-Al_2O_3-MgO slags: Part II-Measurement and prediction of critical cooling rate. J. Non-Cryst. Solids 436, 29–34 (2016).

5. Qin, Y. L., Lv, X. W., Zhang, J., Hao, J. L. & Bai, C. G. Determination of optimum blast furnace slag cooling rate for slag recycling in cement manufacture. Ironmaking Steelmaking 42, 395–400 (2015).

6. Lin, B., Wang, H., Zhu, X., Liao, Q. & Ding, B. Crystallization properties of molten blast furnace slag at different cooling rates. Appl. Therm. Eng. 96, 432–440 (2016).

7. Uhlmann, D. R. & Yinnon, H. Glass: science and technology, vol. 1. New York, U.S.A.: Academic Press; 1983. p. 8 (ch. 1).

8. Rocabois, P., Pontoire, J. N., Lehmann, J. & Gaye, H. Crystallization kinetics of Al_2O_3-CaO-SiO_2 based oxide inclusions. J Non-Cryst Solids 282, 98-109 (2001).

9. Bisio, G. Energy recovery from molten slag and exploitation of the recovered energy. Energy 22, 501-509 (1997).

10. Barati, M., Esfahani, S. & Utigard, T. A. Energy recovery from high temperature slags. Energy 36(9), 5440–5449 (2011).

11. Zhang, H. et al. A review of waste heat recovery technologies towards molten slag in steel industry. Appl. Energy 112, 956–966 (2013).

12. Tsakiridis, P. E., Papadimitriou, G. D., Tsivilis, S. & Koroneos, C. Utilization of steel slag for Portland cement clinker production. J. Hazard. Mater. 152, 805–811 (2008).

13. Iacobescu, R. I. et al. Ladle metallurgy stainless steel slag as a raw material in Ordinary Portland Cement production: a possibility for industrial symbiosis. J. Cleaner Prod. 112, 872–881 (2016).

14. Angulo-Ramírez, D. E., de Gutiérrez, R. M. & Puertas, F. Alkali-activated Portland blast-furnace slag cement: Mechanical properties and hydration. Constr. Build. Mater. 140, 119–128 (2017).

15. Bramer, H. C. Pollution control in the steel industry. Environ. Sci. Technol. 5, 1004–1008 (1971).

16. Stoehr, R. A. & Pezze, J. P. Effect of oxidizing and reducing conditions on the reaction of water with sulfur bearing blast furnace slags. J. Air Pollut. Control Assoc. 25, 1119–1122 (1975).

17. Sun, Y., Zhang, Z., Liu, L. & Wang, X. Heat recovery from high temperature slags: a review of chemical methods. Energies 8, 1917–1935 (2015).

18. Pickering, S. J., Hay, N., Roylance, T. F. & Thomas, G. H. New process for dry granulation and heat recovery from molten blast-furnace slag. Ironmaking Steelmaking 12, 14–20 (1985).

19. Mizuochi, T. et al. Feasibility of rotary cup atomizer for slag granulation. ISIJ Int. 41, 1423–1428 (2001).

20. Purwanto, H., et al. Characteristics of glass beads from molten slag produced by rotary cup atomizer. Mater. Trans. 45, 3286-3290 (2004).

21. Liu, J., Yu, Q. & Guo, Q. Experimental investigation of liquid disintegration by rotary cups. Chem. Eng. Sci. 73, 44-50 (2012).

22. Qin, Y., et al. Dry granulation of molten slag using a rotating multi-nozzle cup atomizer and characterization of slag particles. Steel Res. Int. 84, 852-862 (2013).
23. Wu, J.J., et al. Cold experiment of slag centrifugal granulation by rotary atomizer: Effect of atomizer configuration. Appl. Therm. Eng. 111, 1557-1564 (2017).
24. Mizuochi, T. & Akiyama, T. Cold experiments of rotary vaned-disks and wheels for slag atomization. ISIJ Int. 43, 1469-1471 (2003).
25. Purwanto, H., Mizuochi, T. & Akiyama, T. Prediction of granulated slag properties produced from spinning disk atomizer by mathematical model. Mater. Trans. 46, 1324-1330 (2005).
26. Wang, D., Ling, X. & Peng, H. Simulation of ligament mode breakup of molten slag by spinning disk in the dry granulation process. Appl. Therm. Eng. 84, 437-447 (2015).
27. Liu, J., Yu, Q., Li, P. & Duan, W. Cold experiments on ligament formation for blast furnace slag granulation. Appl. Therm. Eng. 40, 351–357 (2012).
28. Liu, J., Yu, Q., Duan, W. & Qin, Q. Experimental investigation on ligament formation for molten slag granulation. Appl. Therm. Eng. 73, 886–891 (2014).
29. Kashiwaya, Y., In-Nami, Y. & Akiyama, T. Development of a rotary cylinder atomizing method of slag for the production of amorphous slag particles. ISIJ Int. 50, 1245–1251 (2010).
30. Kashiwaya, Y., In-Nami, Y. & Akiyama, T. Mechanism of the formation of slag particles by the rotary cylinder atomization. ISIJ Int. 50, 1252–1258 (2010).
31. Kashiwaya, Y., Akiyama, T. & In-Nami, Y. Latent heat of amorphous slags and their utilization as a high temperature PCM. ISIJ Int. 50, 1259-1264 (2010).
32. Proctor, D. M., et al. Physical and chemical characteristics of blast furnace, basic oxygen furnace, and electric arc furnace steel industry slags. Environ. Sci. Technol. 34, 1576-1582 (2000).

33. Navarro, C., Díaz, M. & Villa-García, M. A. Physico-chemical characterization of steel slag. Study of its behavior under simulated environmental conditions. Environ. Sci. Technol. 44, 5383-5388 (2010).

34. Sun, Y. & Zhang, Z. Utilization of high-temperature slags from metallurgy based on crystallization behaviors. JOM 70, 1274–1281 (2018).

35. Wang, Z. & Sohn, I. A review on reclamation and reutilization of ironmaking and steelmaking slags. J. Sustainable Metal. 5, 127–140 (2019).

36. Tossavainen, M., et al. Characteristics of steel slag under different cooling conditions. Waste Manage. 27, 1335-1344 (2007).

37. Tobo, H. et al. Development of continuous steelmaking slag solidification process suitable for sensible heat recovery. ISIJ Int. 55, 894–903 (2015).

38. Shi, C. & Qian, J. High performance cementing materials from industrial slags—a review. Resour., Conserv. Recycl. 29, 195-207 (2000).

39. Huaiwei, Z. & Xin, H. An overview for the utilization of wastes from stainless steel industries. Resour., Conserv. Recycl. 55, 745-754 (2011).

40. Sun, Y. & Zhang, Z. Energy Saving and Emission Reduction from the Steel Industry: Heat Recovery from High Temperature Slags. In Energy Solutions to Combat Global Warming (pp. 249-280). Springer, Cham (2017).

41. Wang, Z. et al. Effect of Al_2O_3 on the viscosity and structure of CaO-SiO_2-MgO-Al_2O_3-Fe_tO slags. Metall. Mater. Trans. B 46, 537-541 (2015).

42. Yokoyama, K., et al. Separation and recovery of phosphorus from steelmaking slags with the aid of a strong magnetic field. ISIJ Int. 47, 1541-1548 (2007).

43. Kitamura, S. Y., et al. Mass transfer of P_2O_5 between liquid slag and solid solution of 2CaO· SiO_2 and 3CaO· P_2O_5. ISIJ Int 49, 1838-1844 (2009).

44. Du, C. M., et al. Effects of acid and Na_2SiO_3 modification on the dissolution behavior of $2CaO\cdot SiO_2$-$3CaO\cdot P_2O_5$ solid solution in aqueous solutions. ISIJ Int. 56, 1436-1444 (2016).
45. Li, C., Gao, J. & Guo, Z. Separation of phosphorus-and iron-enriched phase from CaO-SiO_2-FeO-MgO-P_2O_5 melt with super gravity. Metall. Mater. Trans. B 47, 1516-1519 (2016).
46. Matsubae-Yokoyama, K., Kubo, H. & Nagasaka, T. Recycling effects of residual slag after magnetic separation for phosphorus recovery from hot metal dephosphorization slag. ISIJ Int. 50, 65-70 (2010).
47. Sun, Y., Zhang, Z., Liu, L. & Wang, X. Integrated carbon dioxide/sludge gasification using waste heat from hot slags: syngas production and sulfur dioxide fixation. Bioresour. Technol. 181, 174-182 (2015).
48. Sun, Y., Zhang, Z., Liu, L. & Wang, X. Two-stage high temperature sludge gasification using the waste heat from hot blast furnace slags. Bioresour. Technol. 198, 364-371 (2015).
49. Sun, Y., et al. Role of steel slags on biomass/carbon dioxide gasification integrated with recovery of high temperature heat. Bioresour. Technol. 223, 1-9 (2017).
50. Sun, Y., et al. Integrated biomass gasification using the waste heat from hot slags: Control of syngas and polluting gas releases. Energy 114, 165-176 (2016).
51. Sun, Y., Chen, J. & Zhang, Z. Biomass gasification using the waste heat from high temperature slags in a mixture of CO_2 and H_2O. Energy 167, 688-697 (2019).
52. Li, P., Yu, Q., Qin, Q. & Lei, W. Kinetics of CO_2/coal gasification in molten blast furnace slag. Ind. Eng. Chem. Res. 51, 15872-15883 (2012).
53. Li, P., Yu, Q., Qin, Q. & Liu, J. Adaptability of coal gasification in molten blast furnace slag on coal samples and granularities. Energy Fuels 25, 5678-5682 (2011).
54. Li, P., et al. CO_2 gasification rate analysis of Datong coal using slag granules as heat carrier for heat recovery from blast furnace slag by using a chemical reaction. Energy Fuels 27, 4810-4817 (2013).

55. Duan, W. et al. Experimental and kinetic study of steam gasification of low-rank coal in molten blast furnace slag. Energy 111, 859-868 (2016).
56. Duan, W. et al. Thermodynamic analysis of hydrogen-rich gas generation from coal/steam gasification using blast furnace slag as heat carrier. Int. J. Hydrogen Energy 39, 11611-11619 (2014).
57. Duan, W. et al. The technological calculation for synergistic system of BF slag waste heat recovery and carbon resources reduction. Energy Convers. Manage. 87, 185-190 (2014).
58. Duan, W. et al. Life cycle and economic assessment of multi-stage blast furnace slag waste heat recovery system. Energy 142, 486–495 (2018).
59. Duan, W., et al. Thermodynamic analysis of synergistic coal gasification using blast furnace slag as heat carrier. Int. J. Hydrogen Energy 41, 1502-1512 (2016).
60. Luo, S., Zhou, Y. & Yi, C. Hydrogen-rich gas production from biomass catalytic gasification using hot blast furnace slag as heat carrier and catalyst in moving-bed reactor. Int. J. Hydrogen Energy 37(20), 15081-15085 (2012).
61. Luo, S., Yi, C. & Zhou, Y. Bio-oil production by pyrolysis of biomass using hot blast furnace slag. Renewable Energy 50, 373-377 (2013).
62. Luo, S. & Feng, Y. The production of fuel oil and combustible gas by catalytic pyrolysis of waste tire using waste heat of blast-furnace slag. Energy Convers. Manage. 136, 27-35 (2017).
63. Li, X. T. et al. Biomass gasification in a circulating fluidized bed. Biomass Bioenergy 26, 171-193 (2004).
64. Channiwala, S. A. & Parikh, P. P. A unified correlation for estimating HHV of solid, liquid and gaseous fuels. Fuel 81, 1051-1063 (2002).
65. Norgate, T., Xie, D. & Jahanshahi, S. Technical and economic evaluation of slag dry granulation. In AISTech2012 Iron & Steel Technology Conference and Exposition 35–46 (2012).

66. Wang, H. et al. Energy–environment–economy evaluations of commercial scale systems for blast furnace slag treatment: dry slag granulation vs. water quenching. Appl. Energy 171, 314–324 (2016).

67. U.S. Geological Survey, 2016 Minerals Yearbook, SLAG—IRON AND STEEL, By Hendrik G. van Oss, 1–9 (2016). https://prd-wret.s3-us-west-2.amazonaws.com/assets/palladium/production/atoms/files/myb1-2016-feste.pdf.

68. IMF, O. (2015). World Economic Outlook: Adjusting to lower commodity prices. https://www.eia.gov/coal/production/quarterly/co2_article/co2.html.

69. Birat, J. P. Global Technology Roadmap for CCS in Industry, Steel Sectoral Report, Contribution to the UNIDO roadmap on CCS - fifth draft, 1–66 (2010).

70. Irlam, L., Policy, S.A. & Economics, A.P.R. Global costs of carbon capture and storage. Global CCS Institute, Melbourne, Australia (2017).

71. Tola, V. & Pettinau, A. Power generation plants with carbon capture and storage: A techno-economic comparison between coal combustion and gasification technologies. Appl. Energy 113, 1461-1474 (2014).

72. Leeson, D., et al. A Techno-economic analysis and systematic review of carbon capture and storage (CCS) applied to the iron and steel, cement, oil refining and pulp and paper industries, as well as other high purity sources. Int. J. Greenhouse Gas Control 61, 71-84 (2017).

73. Tian, S., Jiang, J., Zhang, Z. & Manovic, V. Inherent potential of steelmaking to contribute to decarbonisation targets via industrial carbon capture and storage. Nat. Commun. 9, 4422 (2018).

74. Liu, H. Investigation of coal gasification using blast furnace molten slag as heat carrier. Energy Conservation 6, 41-43 (2004).

75. Bale, C. W. et al. Reprint of: FactSage thermochemical software and databases, 2010–2016. CALPHAD 55, 1–19 (2016).

76. Drissen, P., Ehrenberg, A., Kühn, M. & Mudersbach, D. Recent development in slag treatment and dust recycling. Steel Res. Int. 80, 737–745 (2009).

77. Özbay, E., Erdemir, M. & Durmuş, H. İ. Utilization and efficiency of ground granulated blast furnace slag on concrete properties–A review. Constr. Build. Mater. 105, 423-434 (2016).

78. Yi, H. et al. An overview of utilization of steel slag. Procedia Environ. Sci. 16, 791-801 (2012).

79. Matousek, J. W. The thermodynamic properties of slags. JOM 60, 62-64 (2008).

80. Susa, M., Watanabe, M., Ozawa, S. & Endo, R. Thermal conductivity of $CaO–SiO_2–Al_2O_3$ glassy slags: Its dependence on molar ratios of Al_2O_3/CaO and SiO_2/Al_2O_3. Ironmaking Steelmaking 34, 124-130 (2007).

81. Kang, Y. & Morita, K. Thermal conductivity of the $CaO–Al_2O_3–SiO_2$ system. ISIJ Int. 46, 420-426 (2006).

82. Irfan, M. F., Usman, M. R. & Kusakabe, K. Coal gasification in CO_2 atmosphere and its kinetics since 1948: A brief review. Energy 36, 12–40 (2011).

83. Sutton, D., Kelleher, B. & Ross, J. R. Review of literature on catalysts for biomass gasification. Fuel Process. Technol. 73, 155–173 (2001).

84. Uggetti, E., Ferrer, I., Llorens, E. & García, J. Sludge treatment wetlands: a review on the state of the art. Bioresour. Technol. 101, 2905-2912 (2010).

85. Im-orb, K., Simasatitkul, L. & Arpornwichanop, A. Techno-economic analysis of the biomass gasification and Fischer–Tropsch integrated process with off-gas recirculation. Energy 94, 483-496 (2016).

86. Gao, N., Li, A. & Quan, C. A novel reforming method for hydrogen production from biomass steam gasification. Bioresour. Technol. 100, 4271-4277 (2009).
